# *Chorthippus parallelus* and *Wolbachia*: Overlapping Orthopteroid and Bacterial Hybrid Zones

**DOI:** 10.3389/fgene.2018.00604

**Published:** 2018-12-04

**Authors:** Paloma Martínez-Rodríguez, José L. Bella

**Affiliations:** Departamento de Biología (Genética), Facultad de Ciencias, Universidad Autónoma de Madrid, Madrid, Spain

**Keywords:** *Wolbachia*, *Chorthippus parallelus*, cytoplasmic incompatibility, MLST, bacterial recombination, hybrid zones

## Abstract

*Wolbachia* is a well-known endosymbiotic, strictly cytoplasmic bacterium. It establishes complex cytonuclear relations that are not necessarily deleterious to its host, but that often result in reproductive alterations favoring bacterial transmission. Among these alterations, a common one is the cytoplasmic incompatibility (CI) that reduces the number of descendants in certain crosses between infected and non-infected individuals. This CI induced by *Wolbachia* appears in the hybrid zone that the grasshoppers *Chorthippus parallelus parallelus* (Cpp) and *C. p. erythropus* (Cpe) form in the Pyrenees: a reputed model in evolutionary biology. However, this cytonuclear incompatibility is the result of sophisticated processes of the co-divergence of the genomes of the bacterial strains and the host after generations of selection and coevolution. Here we show how these genome conflicts have resulted in a finely tuned adjustment of the bacterial strain to each pure orthopteroid taxon, and the striking appearance of another, newly identified recombinant *Wolbachia* strain that only occurs in hybrid grasshoppers. We propose the existence of two superimposed hybrid zones: one organized by the grasshoppers, which overlaps with a second, bacterial hybrid zone. The two hybrid zones counterbalance one another and have evolved together since the origin of the grasshopper's hybrid zone.

## Introduction

*Wolbachia* is a well-known endosymbiotic alphaproteobacterium that is able to modify the reproduction (and, in some cases, the behavior) of infected individuals in diverse ways: male feminizing and killing, induced parthenogenesis, cytoplasmic incompatibility (CI), etc. These mechanisms all serve to promote *Wolbachia*'s own, mainly maternal, transmission (Werren et al., 2008; Weinert et al., 2015; Jiggins, 2016). Initially, this infection was considered a kind of parasitism, but subsequent evidence revealed cases of neutral or even beneficial consequences for the host, involving the fecundity of infected individuals, resistance to other infections, etc. (Zug and Hammerstein, 2015; Makepeace and Gill, 2016). Since *Wolbachia* is a strictly cytoplasmic element, it has to establish a sophisticated dialogue not only with the organelles, but also with the nucleus, and even with other infectious organisms, such as the WO phage (LePage et al., 2017). This leads to frequently complex relations with the mitochondria, for example, which may sweep to cytonuclear incompatibilities (Henry and Newton, 2018). The reproductive barriers induced by this bacterium have led it to be considered a speciation agent under some circumstances (Bordenstein et al., 2001; Brucker and Bordenstein, 2012). It is also used as a biological agent against some pests of importance in agriculture and animal husbandry, and its role in some clinical and pathogenic processes cannot be ruled out (LePage and Bordenstein, 2013; Armoo et al., 2017; Ritchie et al., 2018).

Hybrid zones are places where organisms from different species, subspecies, races or, in general, any taxa, meet, interbreed and yield hybrid descendants. They are valuable models in the study of reproductive barriers, gene introgression, speciation and all kinds of evolutionary processes (Hewitt, 1988; Barton and Hewitt, 1989; Harrison, 1993), including those arising in response to climate change (Taylor et al., 2015).

The meadow grasshopper *Chorthippus parallelus* (Orthoptera) forms a Pyrenean hybrid zone that offers an outstanding model for the study of genetic divergence and processes of incipient speciation. During the last Pleistocene Ice Age, this organism retreated into a number of southern European refuges in the Iberian, Italian and Balkan peninsulas of the Mediterranean. Whilst the ice covered northern latitudes, genetic divergence in allopatry gave rise to two isolated subspecies: *C. p. erythropus* (Cpe) in Iberia, and *C. p. parallelus* (Cpp) in the rest of the continent. The ice finally melted after several cycles of advance and retreat, the high mountains of the Alps and Pyrenees being the last places from where it disappeared (Hewitt, 1993, 1996, 2001, 2011). During this time, both taxa kept evolving and diverging with respect to morphological, behavioral, electrophoretic and chromosomal traits (Butlin and Hewitt, 1985a,b; Bella et al., 2007). Genetic differentiation also occurs in certain mitochondrial and nuclear markers (Vazquez et al., 1994; Cooper et al., 1995; Lunt et al., 1998; Korkmaz et al., 2014).

Around 9,000 years ago (and thus 9,000 generations for this grasshopper) these incipient species met in restricted areas under ~2,000 m (the altitudinal limit for this organism) that traverse the Pyrenees. There they formed a hybrid zone, which has been extensively studied from many perspectives over the last 30 years. After generations of mating, gene recombination, selection, etc., the natural hybrids (Cph) between Cpp and Cpe are viable and vigorous (Shuker et al., 2005). They inhabit restricted, narrow areas between the pure subspecies, which are currently too far away from each other to meet again. However, male F1 hybrids obtained in the laboratory are viable but sterile (Hewitt et al., 1987; Bella et al., 1990), in keeping with Haldane's Rule (Coyne, 2018 for a review).

These subspecies and their natural hybrids also differ in the strains of the endosymbiont *Wolbachia* that infect them (Zabal-Aguirre et al., 2010). This infection results in significant cytogenetic and genomic effects, and a partial additional reproductive barrier in the hybrid zone that is sustained by uni- and bidirectional CI (see below). This system constitutes an extraordinary model of coevolution of the genomes of all three taxa (pure Cpp and Cpe, and Cph) with particular *Wolbachia* strains. The result is two overlapping hybrid zones: the aforementioned zone between the grasshopper subspecies, and, as we show here (see below), a second zone in *Wolbachia*, which is indicated by a specific pattern of infection of hybrid grasshoppers and the recombinant *Wolbachia* genomes that specifically appear in them. To our knowledge, this is the first report of a bacterial hybrid zone.

## The Infection of *Chorthippus parallelus* by *Wolbachia*

Dillon et al. (2008) made the first report of infection of Cp by *Wolbachia*. It was soon followed by a cytological survey of *Wolbachia* in squashed and paraffin-embedded Cp tissues by 16S rRNA and wsp surface protein *in situ* hybridization (whole-cell hybridization), which served to confirm the strict cytoplasmic distribution of the infection and the presence of bacteria in female and male gonadal tissues. However, during meiosis in males, there was an accumulation of *Wolbachia* in the pre-spermatic cells, whereby the bacteria aggregated at one pole until they disappeared in mature spermatic cells. Differences in bacterial density among tissues and individuals were also found (Martínez et al., 2009).

A study of around 4,700 individuals from 16 Cp populations inside and outside the hybrid zone, using nested PCR with strain-specific 16S rDNA primers to discriminate between bacterial strains showed that all the locations sampled were infected with *Wolbachia*, but with differences in infection type (B and F strains) and incidence between northern, hybrid and southern (Iberian) populations. This allowed us to distinguish three regional infection patterns associated with the distribution of pure and hybrid Cp individuals. A northern pattern characterized by a low level of B bacterial infection occurred in Cpp populations, while a southern pattern of a high level of infection (F or B or coinfected individuals) was characteristic of Cpe locations. In the hybrid zone, these patterns come together and give rise to a new, characteristic infection pattern that displays a remarkably high degree of co-infection with the two *Wolbachia* strains in hybrid individuals. No statistically significant differences in their proportions were found between the sexes in any of these locations, except for two populations in the hybrid zone that featured a lower than expected proportion of B infection and an excess of uninfected males compared with females (Zabal-Aguirre et al., 2010).

A further survey of the hybrid zone involving 110 crosses that were blind with respect to the presence or absence the strains of *Wolbachia* infection showed no evidence of any effects on female fecundity, except for a minor increase in the proportion of females infected by the F supergroup. However, the analysis of the parents and descendants using the nested-PCR system mentioned above confirmed the significant unidirectional CI, recorded as the relative reduction in embryo production (sh) in crosses involving B and F supergroups, with a sh of 0.355 and 0.286, respectively. A CI with a weaker sh of 0.147 was noted in bidirectional crosses (Zabal-Aguirre et al., 2014). A preliminary study of the gonadal microbiota of 30 Cp individuals from 13 populations, again inside and outside the hybrid zone, ruled out the possible action of other microbial agents that could induce these CIs, even revealing the presence of *Spiroplasma*, another bacterium that, in some cases, can disturb the reproduction of infected individuals (Martínez-Rodríguez et al., 2013). A further study of more than 200 individuals and 17 populations showed that *Spiroplasma* infects distinct *C. parallelus* populations, particularly frequently in those of the Balkan-Alps region. There is a lower frequency of infected individuals in the rest of continental Europe and the Iberian Peninsula, while no infection has been noticed in Southern Iberian populations. However, no interaction has been detected between the two endosymbionts, and there is no evidence of any influence of *Spiroplasma* on *C. parallelus* reproduction (Martínez-Rodríguez, 2013).

A recent analysis involving interannual frequencies of strains of *Wolbachia* across the Cp hybrid zone showed that they change significantly over geographical and temporal scales. By using consecutive years to estimate total *Wolbachia* strain fitness, and computer simulations to discount genetic drift and sampling error, the overall fitness pattern was found to show a negative frequency-dependent trend. This could be induced by natural selection, perhaps reinforced by other intrinsic or extrinsic (ecological) factors affecting the cytonuclear interaction of the infection (Martínez-Rodríguez et al., submitted).

The complexity of these interactions in our model is also indicated by the changes observed in the proportions of infected individuals in the populations during the host life cycle. Testing the infection frequencies several times in the same populations during the life cycle of these grasshoppers revealed significant differences at certain localities during the summer season. These were associated with a threshold temperature that appears to be lethal to the bacteria, but as a result it clearly influences the proportion of incompatible crosses over time in a given population and, consequently, the prevalence and spread of infection in subsequent generations (Martínez-Rodríguez et al., 2014).

The possible influence of *Wolbachia* on certain cytogenetic traits has also been analyzed in a pioneering way in this Cp model system. As expected, hybrid Cp grasshoppers were found to display a significantly higher level of abnormal spermatids (with respect to size, morphology, etc.) than pure Cpp and Cpe. However, when infected by *Wolbachia*, these Cph presented the highest percentage of abnormal spermatids, thereby demonstrating the synergy between the infection and the hybrid condition (Sarasa et al., 2013). As stated above, *Wolbachia* is maternally transmitted and progressively disappears from male meiocytes, as we showed in this system (Martínez et al., 2009), so the long-distance effect on the viability of the spermatids is remarkable. On the other hand, the same study (Sarasa et al., 2013) showed that *Wolbachia* significantly increases the number of chiasma in the meiocytes of infected males. These two situations (the increased frequencies of abnormal spermatids and chiasma in infected individuals) are good examples of a cytonuclear interaction, with the former, implying a degree of incompatibility. These results were also interpreted as secondary effects of a chromatin modification induced by *Wolbachia* under the proposed model of modification/rescue of the chromatin (Beckmann et al., 2017; LePage et al., 2017), and may explain the CI reported in these organisms.

The physical mapping by FISH in the chromosomes of Cp of certain fragments of the *Wolbachia* genome of both supergroups (B and F) that have been laterally transferred into its nuclear genome is an additional cytogenetic finding about this interaction between Cp and *Wolbachia* (Toribio-Fernández et al., 2017). Some of these insertions are subspecies-specific, while others are present in both subspecies. They must be considered as ancestral, given that they do indeed appear in uninfected individuals (Funkhouser-Jones et al., 2015). This additional example of cytonuclear dialogue between the endosymbiotic *Wolbachia* and the Cp genome indicates a close coevolution of the two genomes that we can summarize as specific biogeographical patterns of infection that fit well with the pure Cpp, Cpe or hybrid condition of the grasshoppers (specific bacterial strains preferentially infecting each taxa). This close relationship has to be old, given that certain fragments of the bacterial genome are now canonical parts of the nuclear genome of the grasshopper. However, during this time the genomes must have adapted to live together, resolving conflicts that after generations of counter-balance, still show mismatches like the uni- and bidirectional CI or the increase in frequency of abnormal spermatids in infected males.

## Results

### A Hybrid Bacterial Zone Overlapping That of *Chorthippus parallelus*

A phylogenetic analysis based on *Wolbachia 16S rRNA* gene sequences confirmed that *C. parallelus* are infected by at least four strains of the F supergroup and two strains of the B supergroup (Bella et al., 2010; Zabal-Aguirre et al., 2010; Martínez-Rodríguez et al., 2013). The existence of newly identified genetic markers allows us to characterize with greater precision the genetic diversity, potential recombination phenomena and the geographical distribution of *Wolbachia* through the *Chorthippus parallelus* hybrid zone (see Table S1).

#### How Many *Wolbachia* Strains Infect *C. parallelus*?

Six genes of *Wolbachia* (*coxa, fbpa, ftsz, gatB, hcpA*, and *wsp*) infecting 127 *Chorthippus parallelus* from 21 populations inside and outside the grasshopper hybrid zone (Table 1) were sequenced following the multilocus system typing (MLST system) proposed by Baldo et al. (2006b). As indicated above *Wolbachia* integrated sequences have been detected in *C. parallelus* nuclear genome (Funkhouser-Jones et al., 2015; Toribio-Fernández et al., 2017). However, different evidences rule out that they can introduce false positives in our analyses (see the Supplementary Material section for details).

**Table 1:** Coordinates, altitude, individuals and nomenclature of the sampled populations of *C. parallelus*.

**Populations (as they appear in figures)**	**Population**	**Latitude**	**Longitude**	**Altitude (m)**	***n***
**PYRENEES-HYBRID ZONE**					
HZ France	Arudy (France): ARU	43^°^06^′^ 01^′^′ N	0^°^26^′^ 38^′^′ W	411	5
	Gabas (France): GAB	42^°^53^′^ 60^′^′ N	0^°^25^′^ 60^′^′ W	1,020	4
	L'Hermine (France): HER	42^°^51^′^ 46.8^′^′ N	0^°^23^′^ 30.4^′^′ W	1,209	7
	Soques (France): SOQ	42^°^20^′^ 08^′^′ N	0^°^23^′^ 52^′^′ W	1,396	3
Tourmont	Cabaña Tourmont (France): TOU	42^°^49^′^ 11^′^′ N	0^°^24^′^ 21^′^′ W	1,625	4
Portalet	Portalet (Spain): POR/PORCRU	42^°^48^′^ 03^′^′ N	0^°^24^′^ 54^′^′ W	1,780	17
CM	Corral de Mulas (Spain): CM	42^°^47^′^ 09.4^′^′ N	0^°^23^′^ 34.4^′^′ W	1,569	9
Sallent	Sallent de Gállego (Spain): SAL	42^°^45^′^ 57.5^′^′ N	0^°^20^′^ 33.9^′^′ W	1,343	5
Escarrilla	Escarrilla (Spain): ESC	42^°^43^′^ 54.1^′^′ N	0^°^18^′^ 39.3^′^′ W	1,130	9
**PYRENEES (OTHER)**					
South-Pyrenees/Vielha	Puerto del Cantó (Spain): PCAN	42^°^22^′^ 12.9^′^′ N	1^°^14^′^11.7^′^′ E	1,725	6
	Muna (France): MUN	42^°^53^′^ 53^′^′ N	0^°^37^′^ 48.8^′^′ E	544	2
	Vielha (Spain): VIEL	42^°^40^′^ 25.3^′^′ N	0^°^46^′^ 26.5^′^′ E	1,393	4
**IBERIAN PENINSULA (CENTRAL MOUNTAINS)**					
Center	Navafría (Spain): NAV	40^°^59^′^ 01.95^′^′ N	3^°^49^′^ 00.9^′^′ W	1,780	12
	Becedas (Spain): BEC	40^°^24^′^ 18^′^′ N	5^°^38^′^ 17.2^′^′ W	1,091	3
**IBERIAN PENINSULA (SOUTH)**					
Bubión	Bubión (Spain): BUB	36^°^57^′^ 1.8^′^′ N	3^°^21^′^ 22.8^′^′ W	1,332	3
**IBERIAN PENINSULA (NORTH)**					
North	Basque Country I (Spain): ALA	42^°^58^′^ 41.4^′^′ N	2^°^44^′^ 19.7^′^′ W	625	6
	Basque Country II (Spain): URK	43^°^13^′^ 59.1^′^′ N	2^°^29^′^ 22.3^′^′ W	211	6
**EUROPE**					
Alps	Valdieri (Italy): VAL	44^°^12^′^ 19.74^′^′ N	7^°^22^′^ 47.76^′^′ E	983	4
	Col de L'Arche (France): CLAR	44^°^25^′^ 34.3^′^′ N	6^°^53^′^ 21.6^′^′ E	1,942	2
UK	Epping Forest (England): ENG	51^°^39^′^ 36^′^′ N	0^°^3^′^ 0^′^′ E	102	3
Slovenia	Mokronog (Slovenia): SLO	45^°^56^′^ 37.17^′^′ N	15^°^8^′^ 55.428^′^′ E	242 m	12

Phylogenetic analyses of individual genes were carried out in order to characterize all alleles as belonging to the F or B supergroups (Figure 1; Figures S1–S11). Subsequent MSLT analysis allowed at least 33 different haplotypes or sequence types (ST) to be distinguished based on the combination of five loci. To confirm the other analyses *Wsp* gene was also included (see Figures S9, S10).

**Figure 1:**
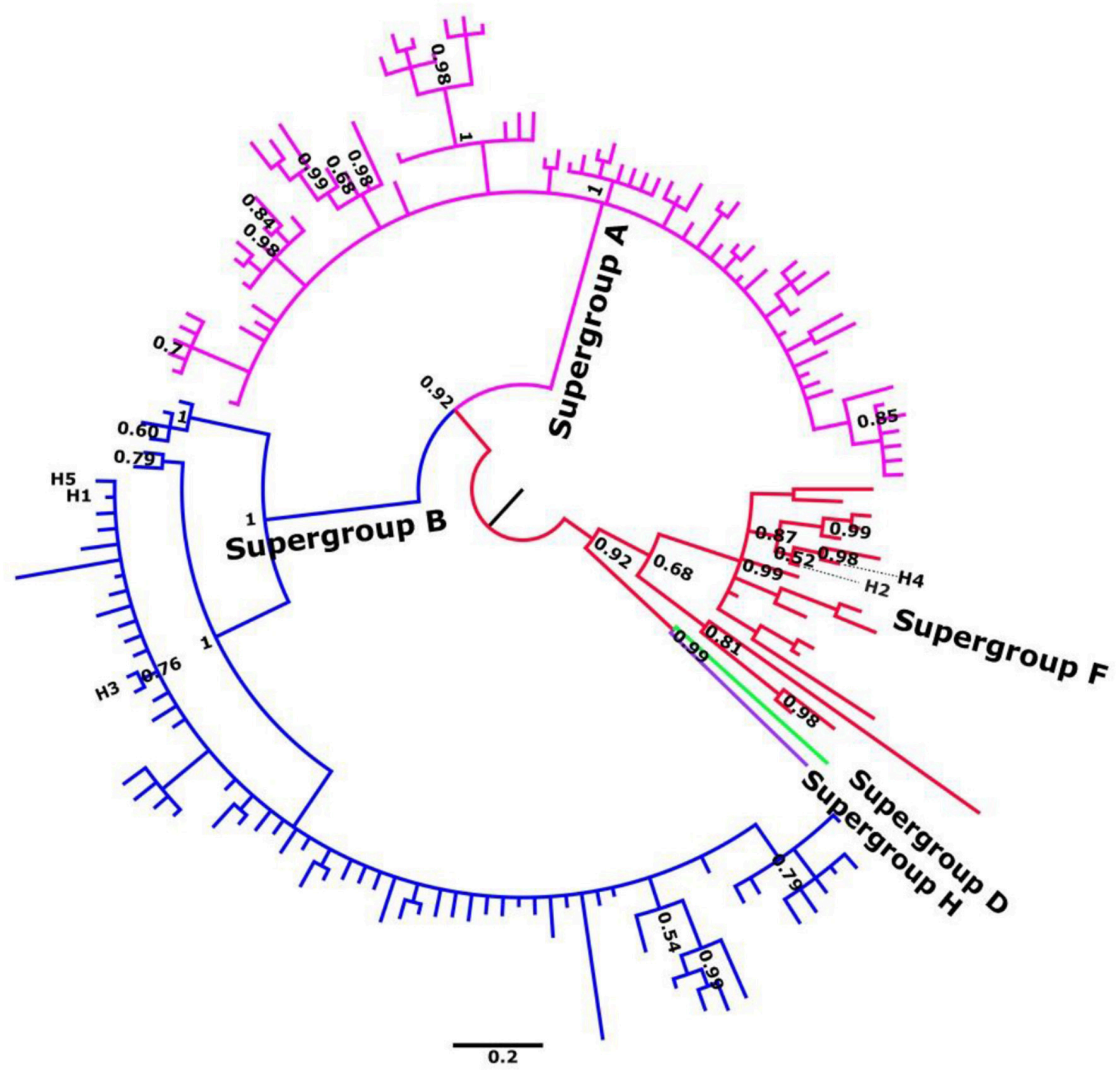
Summary unrooted phylogenetic tree of gatb, obtained by Bayesian inference. Alleles described in *C. parallelus* are named H1–H5. Posterior probabilities are shown at the nodes. Sequence accession numbers are shown in Tables S3–S8.

Nucleotide diversity and other characteristics are summarized in Table 2. In addition, recombination between F and B *Wolbachia* supergroups has been detected in some hybrid populations in the center of the *Chorthippus* hybrid zone (see below). Some recombinants have also been detected in the north of Spain in populations of this grasshopper characterized as hybrid on the basis of chromosomal markers (Bella et al., 2007). By contrast, recombination has not been detected in the pure populations of the grasshopper, even though individuals may have been infected, or even co-infected, by B and F supergroup bacteria.

**Table 2:** Genetic diversity: S, total polymorphic positions; Eta, total frequency of mutations; Hap, frequency of haplotypes; Hd, Haplotype diversity; VarHd, Haplotype diversity variance; Pi, nucleotide diversity; Theta, 4Nu, where N is the effective population size, and u is the mutation rate per nucleotide (or per sequence) and per generation (Tajima, 1983; Nei, 1987).

**Gene**	***n***	**Sites**	**S**	**Eta**	**Hap**	**Hd/VarHd**	**Pi**	**ThetaNuc**	**AvNumDif**	**ThetaG**	**TajimaD**	FuLiD*	FuLiF*	**G+C**	**R (MAXCHI, *p* < 0.01)**
*coxa*	111	402	41	42	6	0.8/0.0002	0.040	0.020	16.251	7.951	3.238**	2.122**	3.090**	0.385	No
*fbpa*	117	429	61	62	5	0.8/0.0001	0.068	0.028	28.579	11.621	4.625***	2.307**	3.943**	0.394	Yes: 3 (83)
*ftsz*	112	435	59	59	5	0.7/0.0002	0.064	0.026	27.801	11.151	4.740***	2.274**	3.965**	0.407	Yes: 1 (34)
*gatB*	114	370	39	39	5	0.7/0.0004	0.047	0.020	17.298	7.346	4.165***	2.092**	3.517**	0.370	No
*hcpA*	115	419	57	57	10	0.9/0.0002	0.056	0.026	23.662	10.719	3.815***	2.057**	3.351**	0.366	No

D, D*, F, and F* statistics test various predictions of the neutral theory of molecular evolution (Tajima, 1989; Fu and Li, 1993) and their significance:

** *p < 0.1*,

*** *p < 0.01*.

*G+C, G+C content; R, Recombination (MAXCHI, Maynard-Smith, 1992)*.

**Figure 2:**
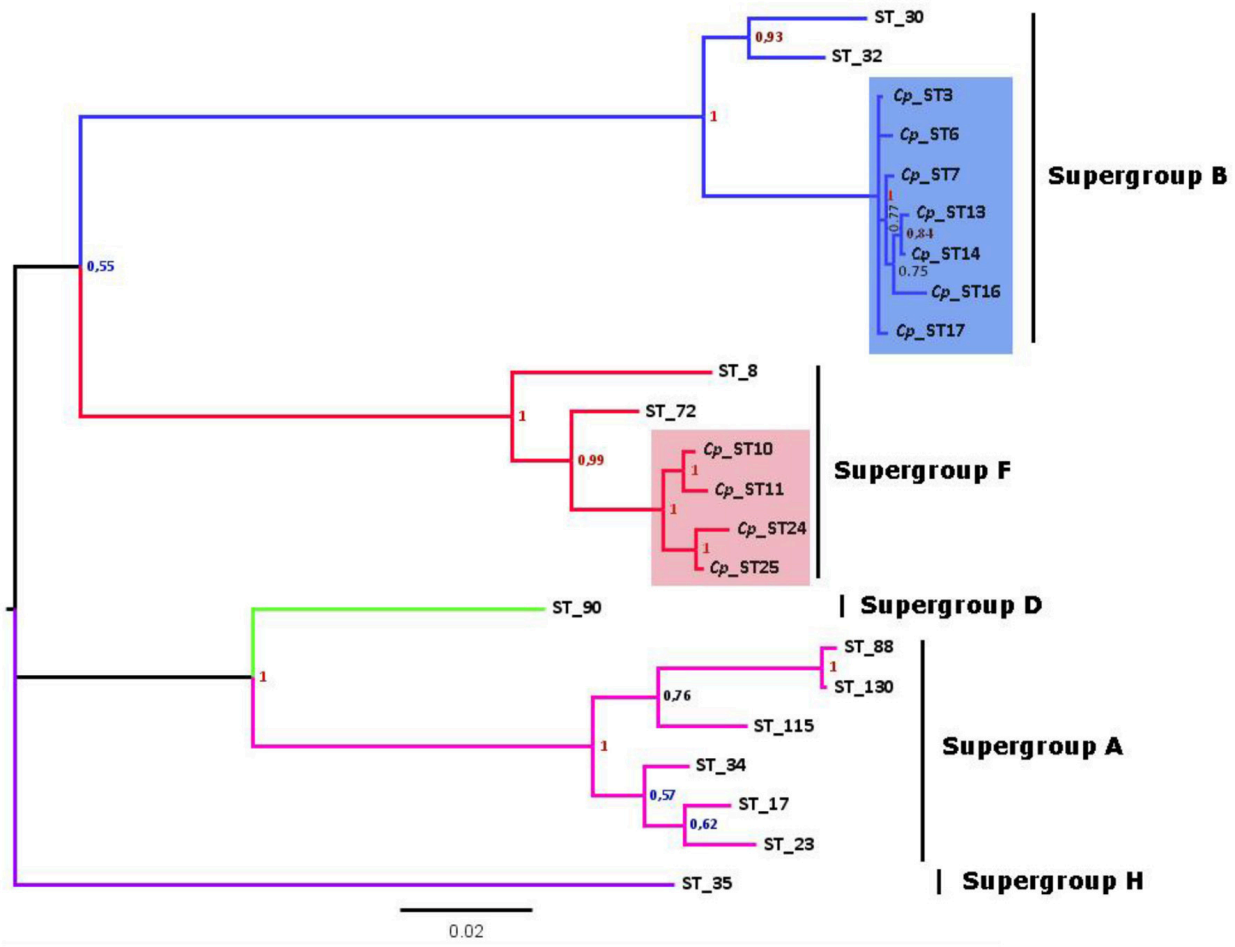
Phylogenetic tree of *Wolbachia* STs detected in *C. parallelus* (marked as Cp, colored squares) excluding recombinants (see Figure 4) obtained by Bayesian inference. The alleles described in this grasshopper bear the prefix Cp_ST. All other STs, named according to the official nomenclature, are available in the MLST database (http://www.mlst.net/). Posterior probabilities are shown at the nodes.

After discarding recombinant STs to avoid artifacts, the phylogenetic tree from concatenated sequences enabled at least 7 strains of *Wolbachia* belonging to the B supergroup and four strains of the F supergroup to be distinguished (Figure 2). This implies a high degree of genetic diversity, given that *Wolbachia* infects two nearby subspecies that have recently diverged (Hewitt, 1993). Furthermore, *Wolbachia* F strains in *C. parallelus* are closely related to one another. The genetic distance between the F strains that infect both subspecies of *Chorthippus parallelus* is shorter than that between the F strains infecting *Chorthippus* and other insects. By contrast, B strains infecting *Chorthippus* are also closely related to other *Wolbachia* strains infecting other Orthoptera, including some species captured in the same populations as *Chorthippus parallelus* (Martínez-Rodríguez, 2013).

#### What Is the Biogeographical Distribution of the *Wolbachia* Strains?

Several analyses have shown important differences in the geographical distribution of the *Wolbachia* strains across the *C. parallelus* populations, all of them showing in practice, the same scenario (Zabal-Aguirre et al., 2010; Martínez-Rodríguez, 2013; Martínez-Rodríguez et al., 2016, see also above**)**. In general, the distribution of the F strains is aligned with the geographical distribution of the two grasshopper subspecies, that is, we find strains of F supergroup *Wolbachia* that are characteristic of Cpp and other strains that have been detected mainly in Cpe. In addition, several strains appear in the *C. parallelus* hybrid zone that have not previously been found in the pure populations. By contrast, we usually detect the same alleles of the B supergroup in Cpp and Cpe populations without distinction. However, we also noted the presence of new B alleles exclusive to *Wolbachia* that infect the hybrid grasshopper populations.

**Figure 3:**
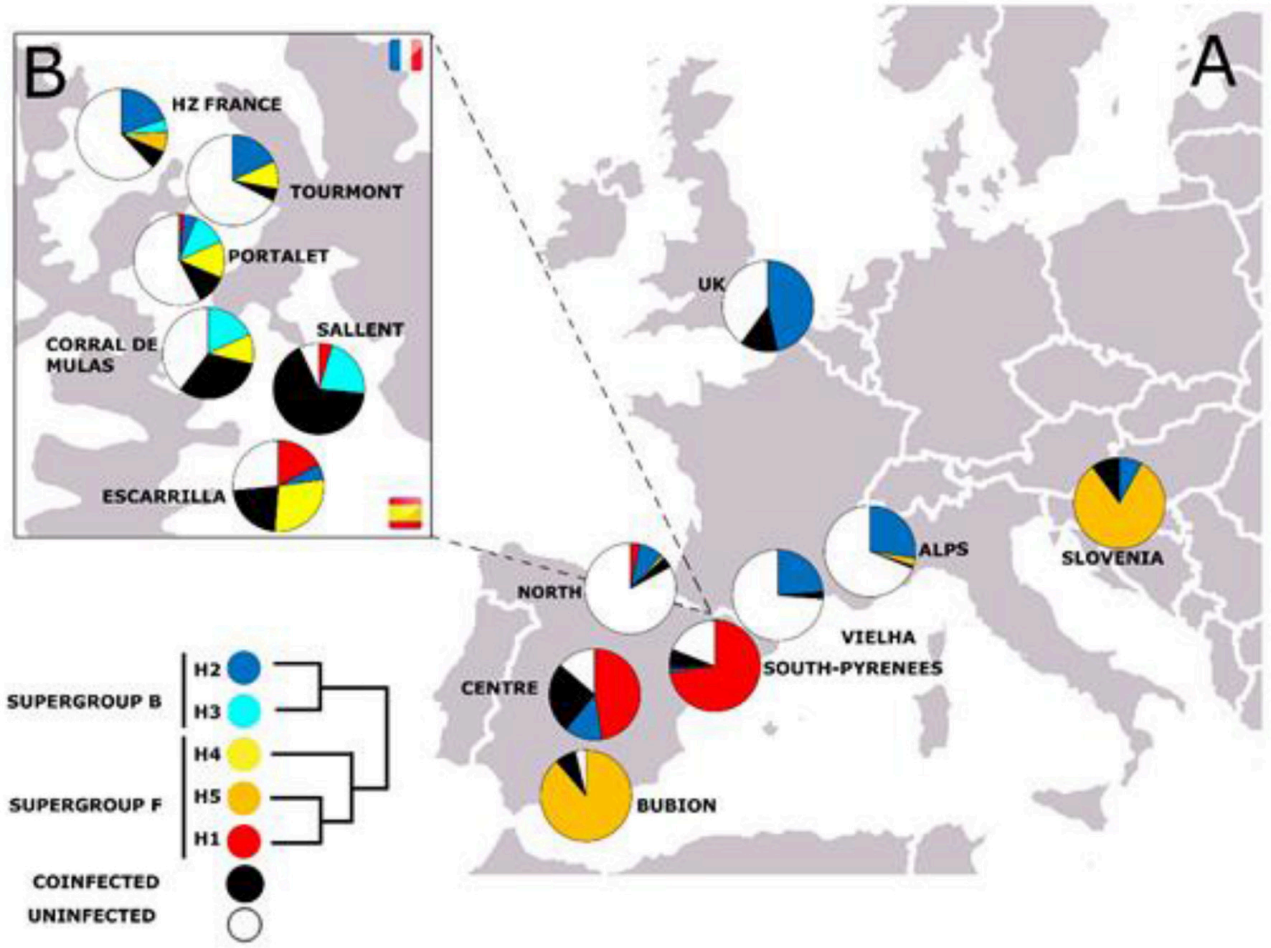
**(A)** Geographical distribution of *fbpA* alleles in the *C. parallelus* populations analyzed. The Pyrenean hybrid zone (Tena Valley, Huesca, Spain) is shown in detail in **(B)**. See Table 1 for details.

**Figure 4:**
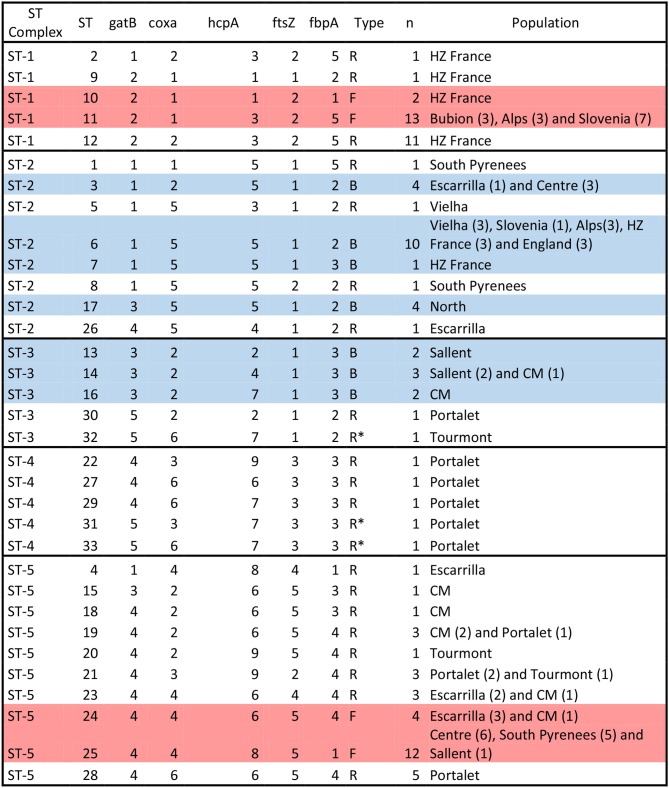
*Wolbachia* ST-complexes and allelic profiles described in *C. parallelus*. Note the classification in three groups: those assigned to supergroups F and B strains (“F” and “B,” respectively) and those in which possible recombination events between these supergroups were observed (“R”). Individuals with no clear assignation are marked as R*. Alleles belonging to the F supergroup (see Figure 1 and Figures S2–S6) are marked in red, while alleles belonging to the B supergroup are marked in blue. STs detected in only one individual should be interpreted with caution, even if the alleles appear in more than one sample. The name of the population and the number of individuals (parenthesis) detected in each population are also indicated.

First, we analyzed all bacterial genetic markers individually. For instance, in the case of the *fbpA* gene, fbpA-1, which belongs to supergroup F, was detected in pure populations from the center of the Iberian Peninsula and in the South Pyrenean populations (see Figure 3). By contrast, fbpA-5 (which is also a member of supergroup F) has mainly been described in pure populations of Cpp (rest of Europe) and in the pure Cpp population of Gabas, on the north side of the hybrid zone (Figure 3). It has also been detected in Bubión (Sierra Nevada, South Spain) and in some populations of the Cantabrian region (hybrid populations, according to Bella et al., 2007). FbpA-4 (also from the F supergroup) has been detected only in the Pyrenean populations of *C. parallelus* and in Cantabrian hybrid populations. In the case of supergroup B, fbpA-2 has been found in Cpp and Cpe populations. However, we have detected some new alleles in the hybrid populations of Sallent, Corral de Mulas and Portalet (see Figures 3, 4 and Supplementary Figures for details**)**. Similar patterns have been observed for all the other genes analyzed (see Figures S12–S16).

Second, we considered all these markers simultaneously, according to the proposed MLST system, in order to gain a global vision of the geographical distribution of *Wolbachia* strains infecting pure and hybrid *C. parallelus*. We classified the different haplotypes or ST into five ST-complexes (each defined as a group of STs sharing a minimum of three alleles) (Figure 4, Figure S17). With respect to the F supergroup, the ST-1 complex has been detected in several pure *C.p. parallelus* populations and in some hybrid populations from the north of the hybrid zone. By contrast, the ST-5 complex, which also belongs to the F supergroup, has been detected in Cpe populations and in some hybrid populations in the south of the hybrid zone.

Considering the B supergroup, the ST2 complex is widely distributed in both subspecies, but the ST3 and ST4 complexes (which include some recombinants and B strains) have been detected mainly in hybrid populations.

**Figure 5:**
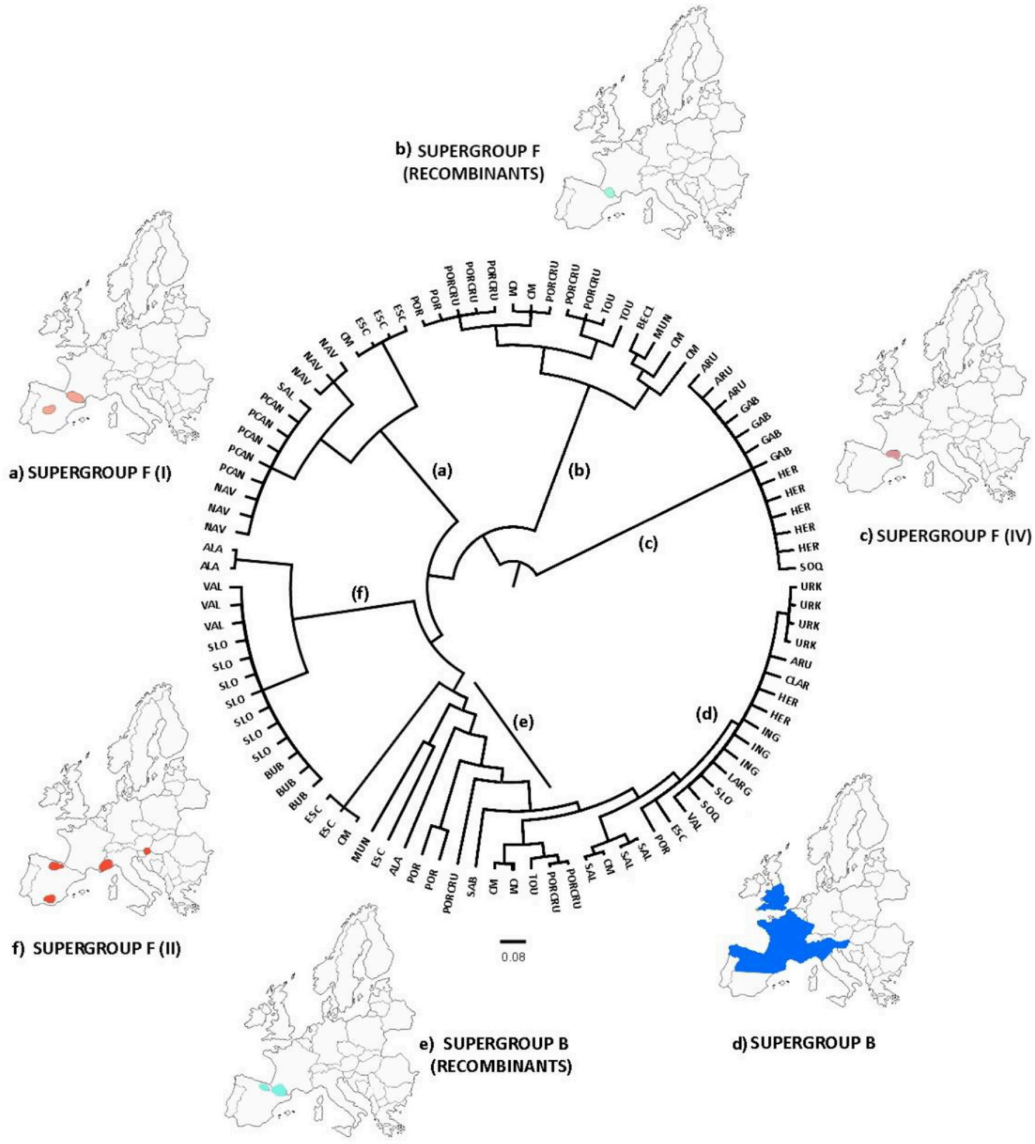
ClonalFrame genealogy. Maps indicate the approximate location of the samples assigned to the major clades, classified with respect to their corresponding F or B supergroup. The analysis distinguished three major clades of supergroup F **(A**,**C**,**F)**, one clade belonging to supergroup B **(D)**, and several recombinant strains **(B**,**E)**. This pattern was consistent with our previous analyses. Acronyms are listed in Table 1.

A new genealogy-based analysis that infers *Wolbachia* microevolution from multilocus sequence data and from a consideration of recombination confirmed the genetic subdivisions in the strains of the F supergroup (Figure 5), while B strains were mainly grouped in the same clade. The genealogies also distinguished the recombinant strains that appear mostly in the grasshopper hybrid zone. The clades also support an association between the genetic and geographic data.

Finally, the less differentiated isolates within geographic populations and the greater differentiation of those between geographic populations suggest isolation-by-distance between the bacterial F strains infecting the two grasshopper subspecies (with the exception of Bubión). An analysis of molecular variation (AMOVA) indicated a geographic division of the F supergroup into: (i) Central Iberian Peninsula and South Pyrenees populations, (ii) Pyrenean hybrid zone populations, (iii) populations on the French side of the hybrid zone, and (iv) non-Iberian populations from the rest of Europe and Bubión (in Spain) (Table 3). These results (except for those for *hcpA*) were also supported by a locus-by-locus AMOVA (Table S1) and an exact test of population differentiation (Rousset et al., 1992) (Table S2). In addition, Mantel tests confirmed that the genetic and geographic distances were correlated (rY1: 0.338, *p* = 0.001). This correlation was stronger when the Bubión data were excluded (rY1: 0.483, *p* = 0.003). This particular geographical distribution could be related to the biogeographical distribution of this grasshopper during the last glaciation, and enables us to infer the origin of *Wolbachia* infection in *C. parallelus* and its role in establishing the hybrid zone.

**Table 3:** Analysis of molecular variance (AMOVA) from five MLST genes for the F supergroup of *Wolbachia* infecting different populations of *C. parallelus*.

**Source of variation**	***df***	**Sum of squares**	**Variance component**	**Percentage of variation**
Between groups	3.00	820.69	13.60	39.80
Between populations within groups	9.00	305.51	4.53	13.25
Between individuals within populations	53.00	850.90	16.05	46.96
Total	65.00	1977.11	34.19	
**Indels:**	**Value**	***P***		
*F*_sc_	0.220	< 0.0001	
*F*_st_	0.530	< 0.0001	
*F*_ct_	0.397	< 0.0001		

## Discussion

### The Co-divergence of the Host and Its Associated Bacteria: *Wolbachia* and *Chorthippus parallelus* Coevolution

The prevalence of *Wolbachia* varies within and among nematode and arthropod taxa, and co-divergence events are known in nuclear and/or mitochondrial hosts genomes and their associated strains (Landmann et al., 2014; Lindsey and Stouthamer, 2017, but see Lefoulon et al., 2016), even including the parallel co-speciation of this bacterium with the common bedbug, *Cimex lectularius* (Balvín et al., 2018).

As described above, new data from the *Wolbachia* MLST system, obtained following Baldo et al. (2006b), confirmed the double infection by F and B supergroups and its distribution in *C. parallelus*. In addition, it revealed the great diversity at the supergroup level. First, these data confirmed the presence of two F bacterial types on both sides of the hybrid zone, as Zabal-Aguirre et al. (2010) suggested. This distribution largely coincides with the biogeography of *C. parallelus*. Second, the new data demonstrated a high degree of diversification of the *Wolbachia* strains infecting grasshopper hybrid populations, including the appearance of new alleles that had presumably arisen from recombination events between *Wolbachia* supergroups.

*Wolbachia* recombination events occur in grasshopper hybrid populations (in which the genomes of Cpp and Cpe met and hybridized), although the F and B supergroups are in contact in many other *C. parallelus* populations, and even co-infect individuals in pure and hybrid populations (Bella et al., 2010; Zabal-Aguirre et al., 2010, 2014). This biogeographical distribution has no simple explanation. It has been proposed that recombination processes between *Wolbachia* strains help the strains develop and adapt rapidly, which is important for their interaction with the host (Werren and Bartos, 2001; Jiggins, 2002). However, the recombination rate between strains is not constant, being respectively less and more common within and between subpopulations. Thus, those strains adapted to a particular host have limited levels of recombination compared with those that exceed the limit of the subpopulation, for example, by coming into contact with another host. These would have higher rates of recombination (Klasson et al., 2009). This hypothesis could explain the distribution of *Wolbachia* in the *C. parallelus* hybrid zone. After thousands of generations of coevolution between *Wolbachia* strains and pure host genomes, the formation of the hybrid zone placed the *Wolbachia* strains in contact with a new host genome (the hybrid genome) and accelerated the recombination process. This might explain why our analysis exclusively detects recombining strains (bacterial hybrid strains) that infect grasshopper hybrid populations.

In addition, we do not know the effect of recombination on the bidirectional CI between strains. Recombination could reduce it, or maybe favor the appearance of a new, mixed bacterial strain because of the host's genetic background in this area, in contrast to the pure populations on both sides of the Pyrenees. Further studies should be carried out to confirm this, but we note the smaller relative reduction in embryo production in the bidirectional crosses in the hybrid zone (Zabal-Aguirre et al., 2014; see above).

### Origin of *Wolbachia* Infection in *C. parallelus* and Its Effects on the Origin of the Hybrid Zone

**Figure 6:**
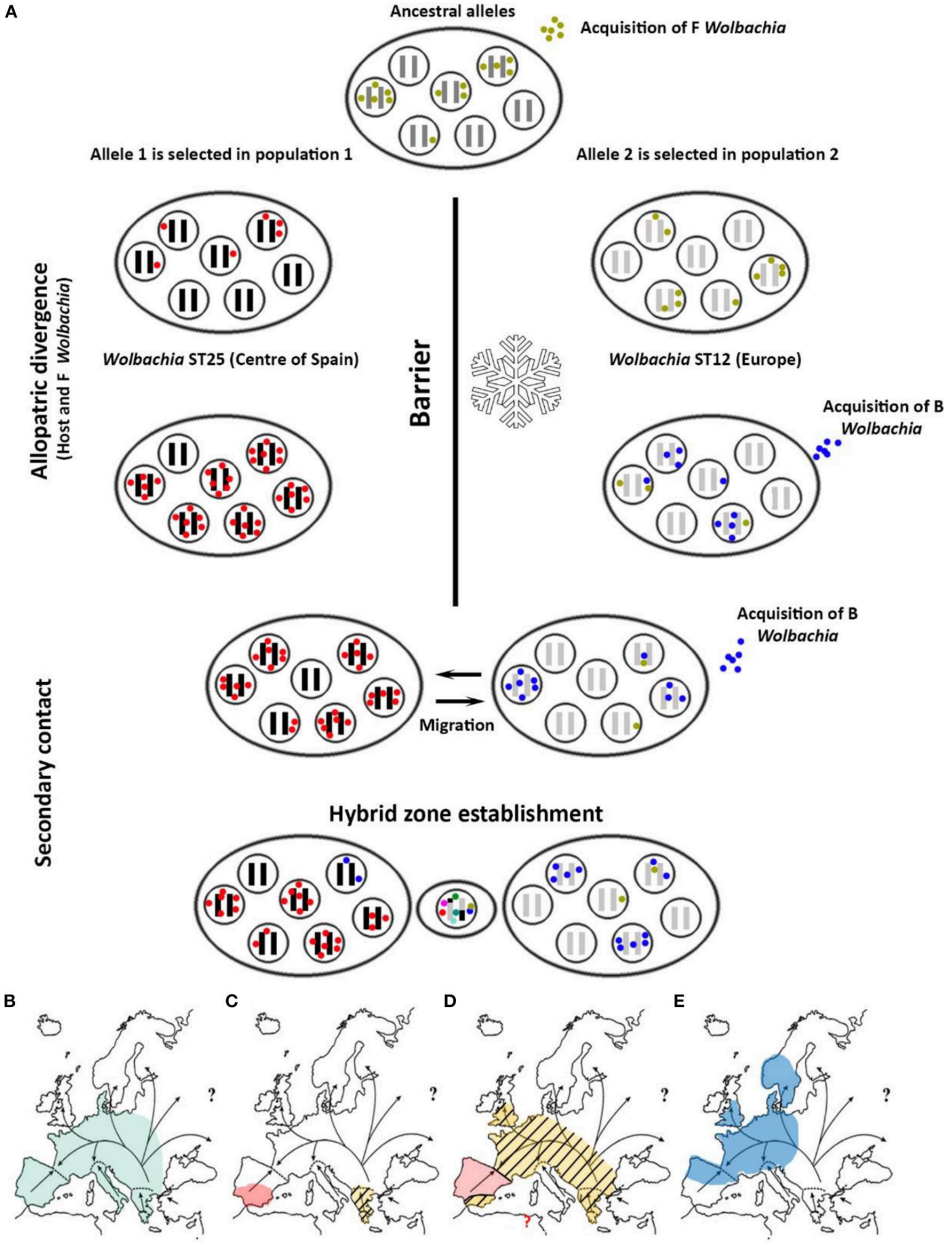
**(A)** Proposed hypothesis for the origin of *Wolbachia* infection in *C. parallelus*. Each ellipse represents a population. Inner circles represent individuals. Black and gray bars indicate the host genome, while the colored dots show the bacterial type infecting the individual. The hybrid zone would be established simultaneously with the appearance of recombinant genomes in the host, and considerable bacterial diversity, induced by recombination. **(B)** Spatial representation of the expansion of the infection: the arrows indicate the population expansion of *C. parallelus* (modified from Hewitt, 2001) after the retreat of the glacial ice. Before the last glaciation the infection of *Wolbachia* by the F supergroup was homogeneous. **(C)** During the last glaciation, *C. parallelus* and F *Wolbachia* diverged in allopatry. **(D)** After the ice disappeared, the pattern of expansion of the F infection coincided with that of the migration of its host. **(E)** Recently, B infection has been transmitted (also horizontally) in various European populations.

The new genetic data presented here help us infer the origin of *Wolbachia* infection in *C. parallelus*, and its role in the dynamics of the hybrid zone. Previous studies showed that the grasshopper subspecies diverged as a result of their geographic isolation in allopatry (Hewitt, 1993, 1999, 2001, 2011; Serrano et al., 1996) during the last glaciation. Recent cytogenetic data suggest that bacterial F strains and the ancestral *C. parallelus* came into contact before the hybrid zone was formed (Toribio-Fernández et al., 2017). In addition, the phylogeographical distribution of *Wolbachia* suggests a strong correlation between *Wolbachia* and its host's biogeography. First, each subspecies of *C. parallelus* is infected mainly by a specific F strain of *Wolbachia* (Figure 6). Second, both F strains, which infect the two grasshopper subspecies, are more closely related than are other F strains that infect other hosts (Baldo et al., 2006a, 2007), as our phylogenetic analysis confirmed. So, even if co-divergence from allopatric hosts is apparently less common than horizontal transmission from other species (Raychoudhury et al., 2009), all the data suggest that F strains co-diverge with *C. parallelus* during host isolation. After the retreat of the ice, grasshopper populations from the Iberian Peninsula colonized the Pyrenees, meeting Cpp coming from the Balkans, as suggested by Lunt et al. (1998). Genetic incompatibilities between the host and bacterium accumulated during the divergence, together with the phenomenon of unidirectional IC, thereby influencing the formation of the current grasshopper hybrid zone. By contrast, the origin of the current F infection in Bubión is not known. The presence of the same strain that we detected in other European populations, as well as some exclusive cytogenetic markers (Bella et al., 2007), is intriguing, and more analyses are required to develop a hypothesis.

By contrast, the B supergroup could have been recently acquired as a result of rapid expansion of the infection from other taxa (horizontal transmission). In fact, further data have revealed closely related B strains in other orthopteroids that share the habitat with *C. parallelus* (Martínez-Rodríguez, 2013). This hypothesis is also supported by the homogeneity of the bacterial B strains located in distant populations, as well as the estimates of divergence between the strains detected in the hybrid zone and the rest of Europe. In addition, the lack of B infection in some populations, like Bubión in southern Spain, also suggests a recent spread of infection from continental Europe: the isolation of these populations and their geographical location has not yet resulted in an established infection. Loss of an ancestral B infection in this population seems less plausible to us, given the strain's aforementioned homogeneity.

Our results indicate that *Wolbachia* was present in the *Chorthippus* hybrid zone, where it has exerted its influence since the zone originated. This is therefore a relevant factor to be considered in the study of this model of incipient speciation.

## Overlapping Orthopteroid and Bacterial Hybrid Zones?

Our data suggest the existence of a bacterial hybrid zone that is superimposed on the grasshopper's hybrid zone. The two zones would obviously be dynamically dependent, maintained by a delicate balance of multiple interactions. We are reminded that the *C. parallelus* hybrid zone was originally described as a secondary contact zone, maintained by the balance between dispersal and the presumed reduced fitness of the natural hybrids. This is supported by the close adherence to Haldane's rule (whereby heterogametic males are sterile) of F1 laboratory hybrids of pure individuals of the two subspecies, their mating behavior and the homogamy detected in studies of female sperm preference (Shuker et al., 2005). In the presumed genomic conflict between the two grasshopper subspecies, *Wolbachia* plays an additional role in reinforcing the reproductive barrier between them, as demonstrated by the unidirectional and bidirectional CI that it induces in field-collected hybrids (Zabal-Aguirre et al., 2014) and the increase in abnormal spermatid production (Sarasa et al., 2013). All of this reveals a complex scenario of specific coevolution of the endosymbiont and its host in continental Europe (pure *C.p. parallelus*), Iberia (pure *C.p. erythropus*) and the hybrid zone (hybrid *C. parallelus*), whereby each area and organism displays peculiarities and specific coadaptations. For this system to function successfully, several processes need to be involved, including genome shock. Genome hybridization could accelerate changes at the genetic and epigenetic levels, and we cannot rule out the possibility that these may affect the endosymbiont-host relationship.

Overall, by simultaneously considering the host and endosymbiont genomes, our findings suggest the existence of three main genetic groups: Cpe-IberianWolF, Cpp-WolB, and Hybrid-EuropeanWolF. Preliminary microsatellite data confirm the high degree of genetic *C. parallelus* diversification in hybrid populations and differentiate the same three population groups in the hybrid zone (Sarasa, 2013). *Wolbachia* strains coincide with this distribution, including the new hybrid bacterial strains that exclusively infect hybrid grasshopper populations, thereby indicating the co-divergence and coevolution of the bacterial strains with their hosts, but also the result of their genomic conflict and the delicate balance of cytonuclear compatibilities and incompatibilities (Bhattacharya and Newton, 2017).

## Data Availablity Statement

DNA sequences: GenBank database under accession numbers KM078849-KM078883.

## Author Contributions

All authors listed have made a substantial, direct and intellectual contribution to the work, and approved it for publication.

### Conflict of Interest Statement

The authors declare that the research was conducted in the absence of any commercial or financial relationships that could be construed as a potential conflict of interest.
