# Supplementary material for: Chorthippus parallelus and Wolbachia: Overlapping Orthopteroid and Bacterial Hybrid Zones
Source: Front Genet. 2018 Dec 4;9:604. doi: 10.3389/fgene.2018.00604 (PMC6288197; doi:10.3389/fgene.2018.00604)
Supplement: Supplementary file 1 [file Data_Sheet_1.docx]

# ***Chorthippus parallelus* and *Wolbachia*: overlapping orthopteroid and bacterial hybrid zones**

Paloma Martínez-Rodríguez and José L. Bella^*^

Departamento de Biología (Genética), Facultad de Ciencias, Universidad Autónoma de Madrid. 28049 Madrid, Spain.

***Correspondence:**Corresponding Author
bella@uam.es

**Key words: *Wolbachia*, *Chorthippus parallelus*, cytoplasmic incompatibility, MLST, bacterial recombination, hybrid zones.**

**Supplementary material:**

**Material and methods**

*Field collections*

127 *Chorthippus parallelus* individuals were collected from 21 European and Iberian populations, grouped as indicated in Table 1, to analyse *Wolbachia* diversity. Gonads were dissected and fixed in 100% ethanol.

*DNA extraction,* Wolbachia *detection and sequencing*

DNA was extracted from whole fixed ovaries and testes, as described elsewhere (Martínez-Rodríguez et al., 2013a,b). *Wolbachia* was detected by PCR amplification of a *Wolbachia 16S rRNA* gene, using *Wolbachia-*specific primers (Zabal-Aguirre et al., 2010), followed by a second, nested PCR amplification using strain-specific primers (Martínez-Rodríguez et al., 2013a,b) (Table 2). **PCR and nested PCR were performed following the highest quality standards, including positive and negative controls, and further PCR amplification of these negative controls to guarantee to avoid false positives**. 10 µl of each amplification product were electrophoretically separated on 1% agarose gels, which were stained with 0.5 mg/ml ethidium bromide and visualised under UV light (UVIdoc, Uvitec Cambridge). **Sequencing of individual reactions have been repeatedly performed, always resulting in the expected *Wolbachia* strain as, for example, in Zabal-Aguirre et al. 2010; Martínez-Rodríguez et al. 2013a; Funkhouser et al. 2015 and Toribio et al. 2017**

We characterised the *Wolbachia* strains using the MLST and *wsp* (*Wolbachia* surface protein) gene characterisation systems (Baldo et al*.* 2006a). The *GatB*, *coxA*, *hcpA*, *ftsZ*, *fbpA* and *wsp* genes in singly infected individuals (as previously tested by *16S rRNA* gene primers) were characterised in this way, while co-infected individuals were discarded. These genes were amplified using previously described methods (Baldo et al., 2006a) with slight modifications: PCR reactions were performed in 50-µl volumes containing 2 mM of MgCl_2_, 0.2 mM of dNTP, 30 pmoles of each primer, 1.25 U of *Taq* BIOTAQ™ DNA polymerase (Bioline) and 2 µl of DNA solution (50 ng/µl). 10 µl of each amplification product were electrophoretically separated on a 2% agarose gel, which was stained and visualised as described above. Amplified genes were purified by ExoSAP-IT (GE Healthcare) and sequenced with Stabvida; www.stabvida.com**¡Error! Referencia de hipervínculo no válida.**). The MLST and *wsp* sequences generated in this study have been deposited in the GenBank database under accession numbers KM078849-KM078883.

**We discard false positives due to the *Wolbachia* insertions reported by Funkhouser-Jones et al. (2015) and Toribio et al. (2017), mainly because these *Wolbachia* insertions also appear in uninfected individuals (so characterized by the nested PCR system here utilised). If the insertions were able to produce false-positives, we would not detect uninfected individuals, given that the insertions seem to be in the *Chorthippus* genome after generations (clearly before the divergence of *C.p. parallelus* and *C.p. erythropus*) and currently present in all *Chorthippus parallelus* individuals. Furthermore, we would expect differences in the amplicons size PCR obtained between integrated fragments and those amplified from cytoplasmic infection. To limit this risk, each PCR band was isolated before sequencing and only those fitting with the right size were used (the appearance of other bands or DNA smears was really unusual). Moreover, DNAs were extracted from gonad tissue in order to increase the cytoplasmic bacteria/insertions ratio. Previous studies confirm a high level of *Wolbachia* in the grasshopper gonads (Martínez et al., 2009). This reduces the probability of amplification of *Wolbachia* insertions and improves the reliability of the Sanger sequencing. Furthermore, to distinguish the sequences belonging to *Wolbachia* infection and those integrated into the host nucleus all sequences were translated into protein. This serves to detect frameshift mutation, stop codons and indels. Our previous studies in another grasshopper, *Podisma pedestris*, confirm that most of the *Wolbachia* nuclear insertions show these mutations, due to the absence of evolutionary constraints after integration (non-translated sequences) (Martínez-Rodríguez *et al.*, data non published).**

**Phylogenetic analysis**

Maximum likelihood (ML) and Bayesian phylogenetic trees were generated to classify *Wolbachia* strains. The maximum phylogenetic tree was inferred using RaxML 7.0.4 (Stamatakis, 2006; Stamatakis et al., 2007). The GTR+I+G model (general time-reversible model, including gamma and proportion-invariant corrections) was selected for all genes. ML heuristic searches were performed using 100 random taxon addition replicates. ML bootstrap support was determined from 100 bootstrap replicates. Bayesian likelihood was inferred using a Markov Chain-Monte Carlo (MCMC) variant run in the MrBayes 3.2.1 program (Ronquist and Huelsenbeck, 2003). Phylogenies based on single and concatenated MLST genes and *wsp* were reconstructed. JModeltest (Posada, 2008) was used to distinguish the appropriate model of evolution, the best likelihood score being chosen on the basis of the AIC criteria (Akaike, 1974). The selected models were GTR+I+G for concatenated MLST, *ftsZ* and *gatB*; GTR+G (general time-reversible model, including gamma correction) for *coxA* and *hcpA* and *wsp*; and HKY+I+G (the Hasegawa, Kishino and Yano model, including gamma and the proportion of invariant corrections) for the *16S rRNA* gene. Bayesian analysis was carried out for 10^6^ generations with a sample frequency of 100. The first 25% of trees were considered as burn-in and thus discarded.

Differences between alternative tree topologies were evaluated using the AU (approximately unbiased), KH (Kishino-Hasegawa) and SH (Shimodaira-Hasegawa) tests (Kishino and Hasegawa, 1989; Shimodaira and Hasegawa 1999; Shimodaira, 2002) using CONSEL (Shimodaira and Hasegawa 2001) and TREE-PUZZLE (Schmidt et al., 2002).

For each locus, the level of nucleotide diversity per site and the number of variable sites or Ka/Ks were estimated using DnaSP software (Librado and Rozas, 2009).

**Recombination analysis**

Alignments of individual and concatenated genes with and without outgroups were screened for significant levels of recombination using RDP4 v4.16 (Martin et al., 2010a). The analysis involved several tests including GENECONV (Padidam et al., 1999), MAXCHI (Maynard Smith, 1992) and Chimaera (Posada and Crandall, 2001). A Bonferroni correction was applied and significance was concluded for values of p < 0.01.

**Strain characterisation**

Following the MLST system (Baldo et al., 2006b; Maiden et al., 1998), we recognised a *Wolbachia* strain or sequence type (ST) as being different on the basis of combinations of five alleles. These combinations were employed as molecular markers and for detecting recombination. Furthermore, strains sharing at least three alleles were considered to be an ST complex, a group of evolutionarily related haplotypes. This analysis was carried out using START2 (Jolley et al., 2001). The *wsp* system was employed as a complementary approach for strain characterisation (Baldo et al., 2005). Recurrence and geographical correlation (see Results) enabled PCR-associated sequencing errors to be excluded.

**Inference of bacterial microevolution using multilocus sequence data**

We inferred *Wolbachia* microevolution using ClonalFrame, which allowed us to identify the clonal relationships between strains, and to estimate recombination events that had disrupted clonal inheritance (Didelot and Falush, 2007). We performed five separate runs, executing 250,000 MCMC iterations for each, discarding the first 100,000 iterations as burn-in.

**Biogeographical analysis**

An AMOVA of the ST frequencies detected in each population was carried out based on the estimated supergroup frequencies (some data published: Bella et al., 2010; Zabal-Aguirre et al., 2010) and the genetic distance between haplotypes (calculated as the Tamura–Nei distance). Locus-by-locus AMOVA and an exact test of population differentiation were also carried out. In addition, we tested the correlation between genetic and geographical distances with Mantel tests. Geographical distance was estimated using the Geographical Distance Matrix Generator v.1.2.3. All analyses were done using Arlequin (Excoffier et al., 2005).

**Supplementary Figures**


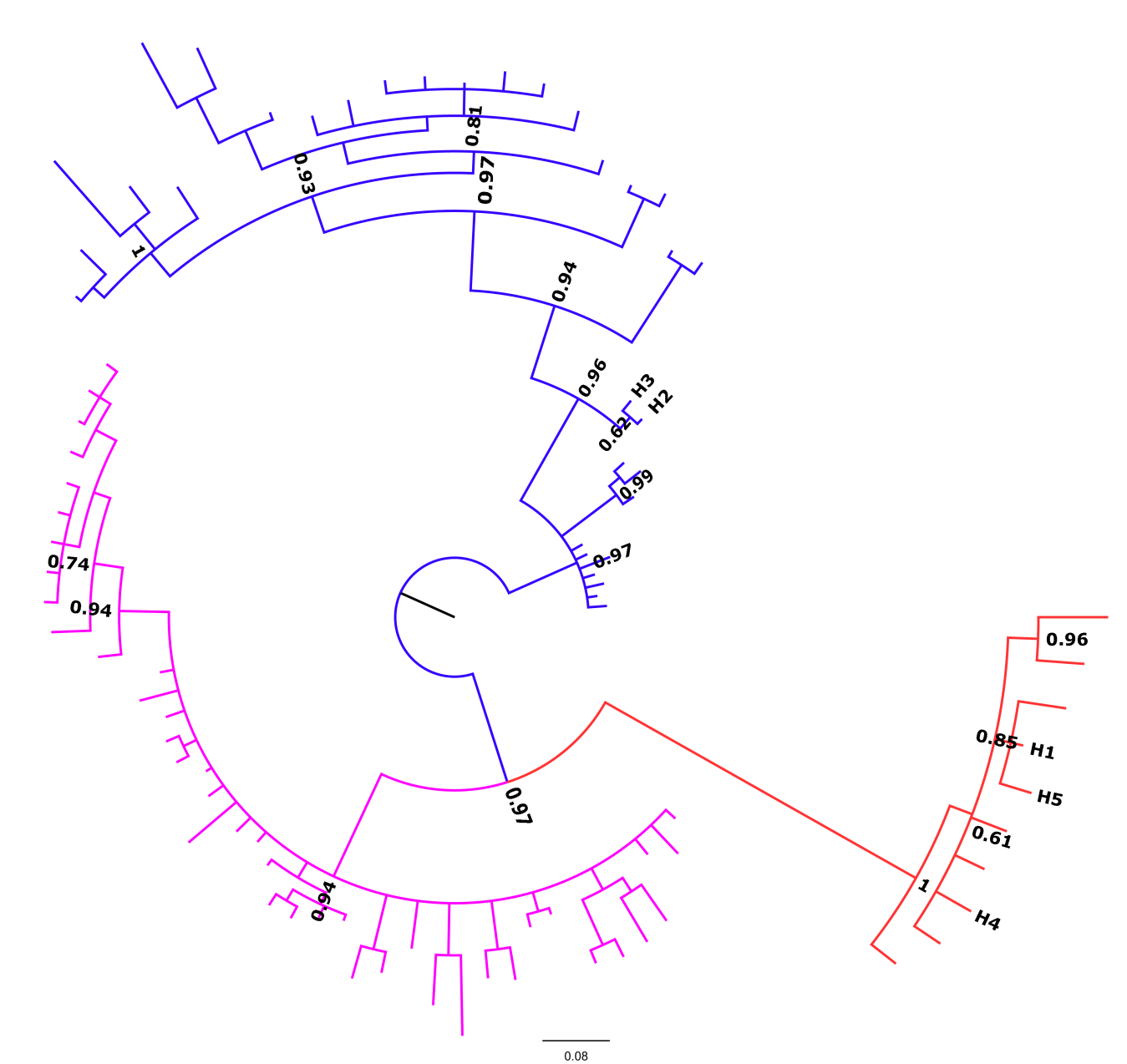


Figure S1: Unrooted phylogenetic tree of *fbpA*, obtained by Bayesian inference. Alleles described in *C. parallelus* are named H1 to H5. Posterior probabilities are shown in the nodes. The colour code indicates supergroups A (pink), B (blue), D (green), F (red) and H (purple). Posterior probabilities are shown in the nodes.


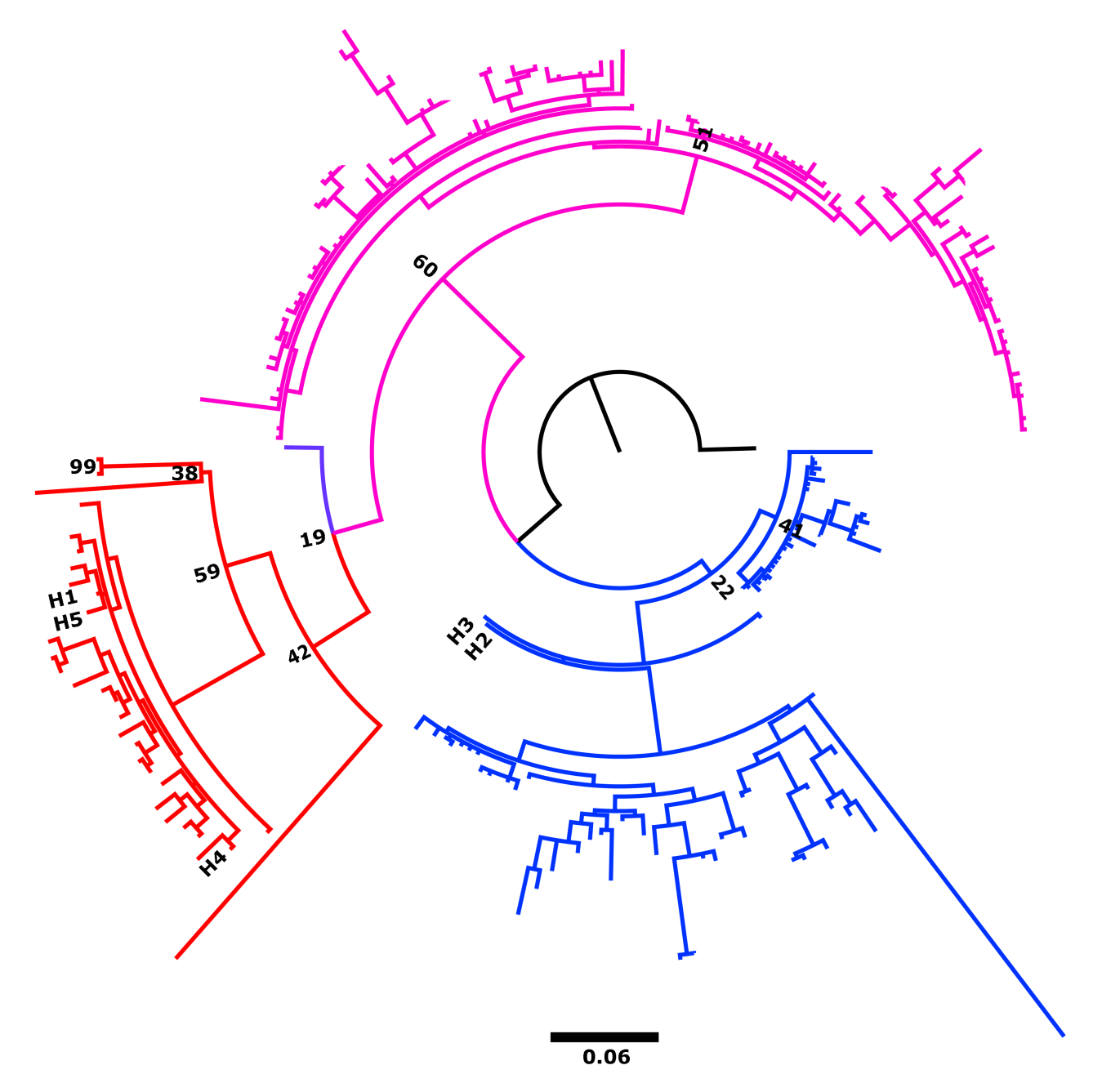


Figure S2: Unrooted phylogenetic tree of *fbpA*, obtained by ML. Alleles described in *C. parallelus* are named H1 to H5. Posterior probabilities are shown in the nodes. The colour code indicates supergroups A (pink), B (blue), D (green), F (red) and H (purple). Bootstrap values are shown in the nodes.


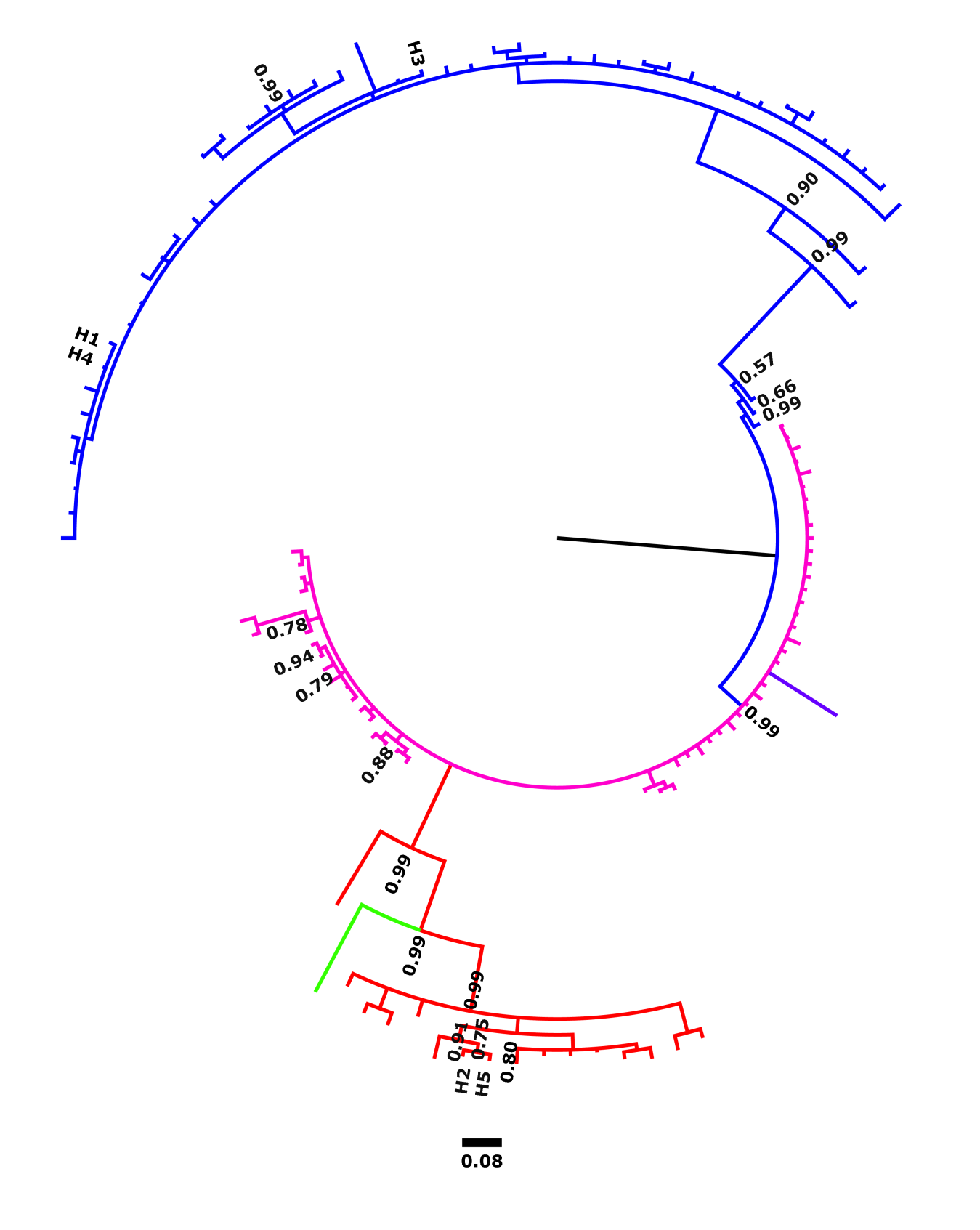


Figure S3: Unrooted phylogenetic tree of *ftsZ*, obtained by Bayesian inference. Alleles described in *C. parallelus* are named H1 to H5. Posterior probabilities are shown in the nodes. The colour code indicates supergroups A (pink), B (blue), D (green), F (red) and H (purple). Posterior probabilities are shown in the nodes.


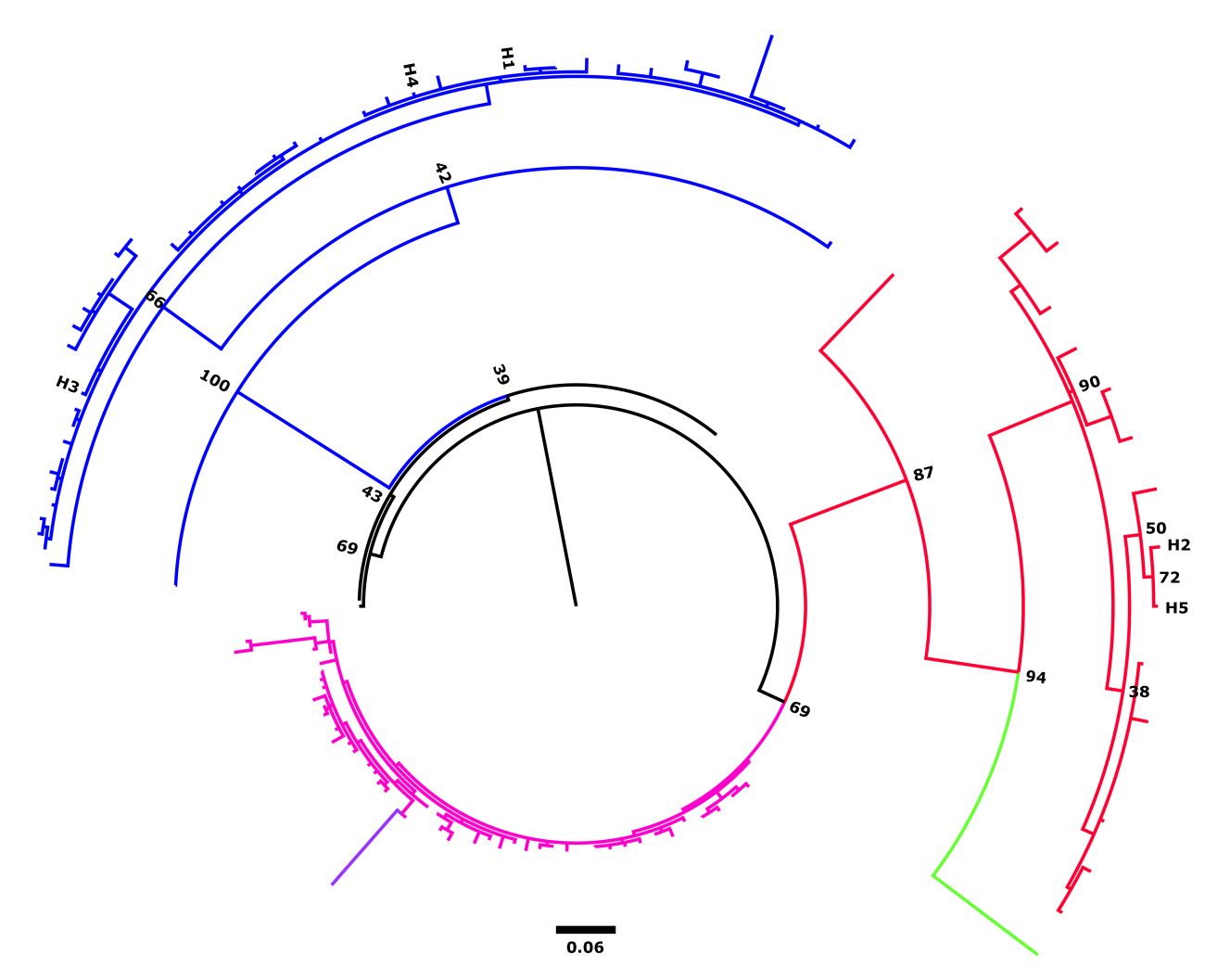


Figure S4: Unrooted phylogenetic tree of *ftsZ*, obtained by ML. Alleles described in *C. parallelus* are named H1 to H5. Posterior probabilities are shown in the nodes. The colour code indicates supergroups A (pink), B (blue), D (green), F (red) and H (purple). Bootstrap values are shown in the nodes.


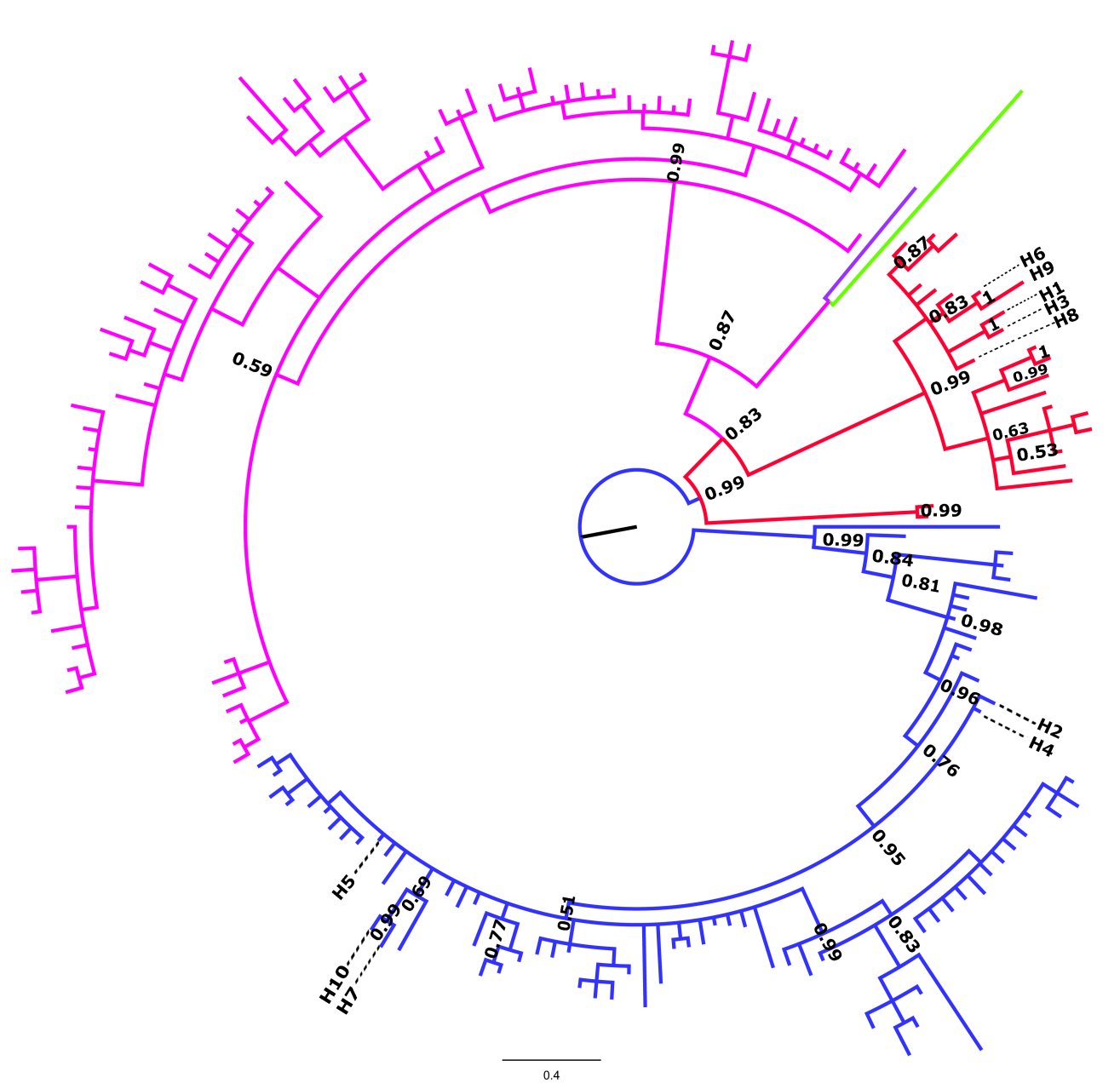


Figure S5: Unrooted phylogenetic tree of *hcpA*, obtained by Bayesian inference. Alleles described in *C. parallelus* are named H1 to H10. Posterior probabilities are shown in the nodes. The colour code indicates supergroups A (pink), B (blue), D (green), F (red) and H (purple). Posterior probabilities are shown in the nodes.


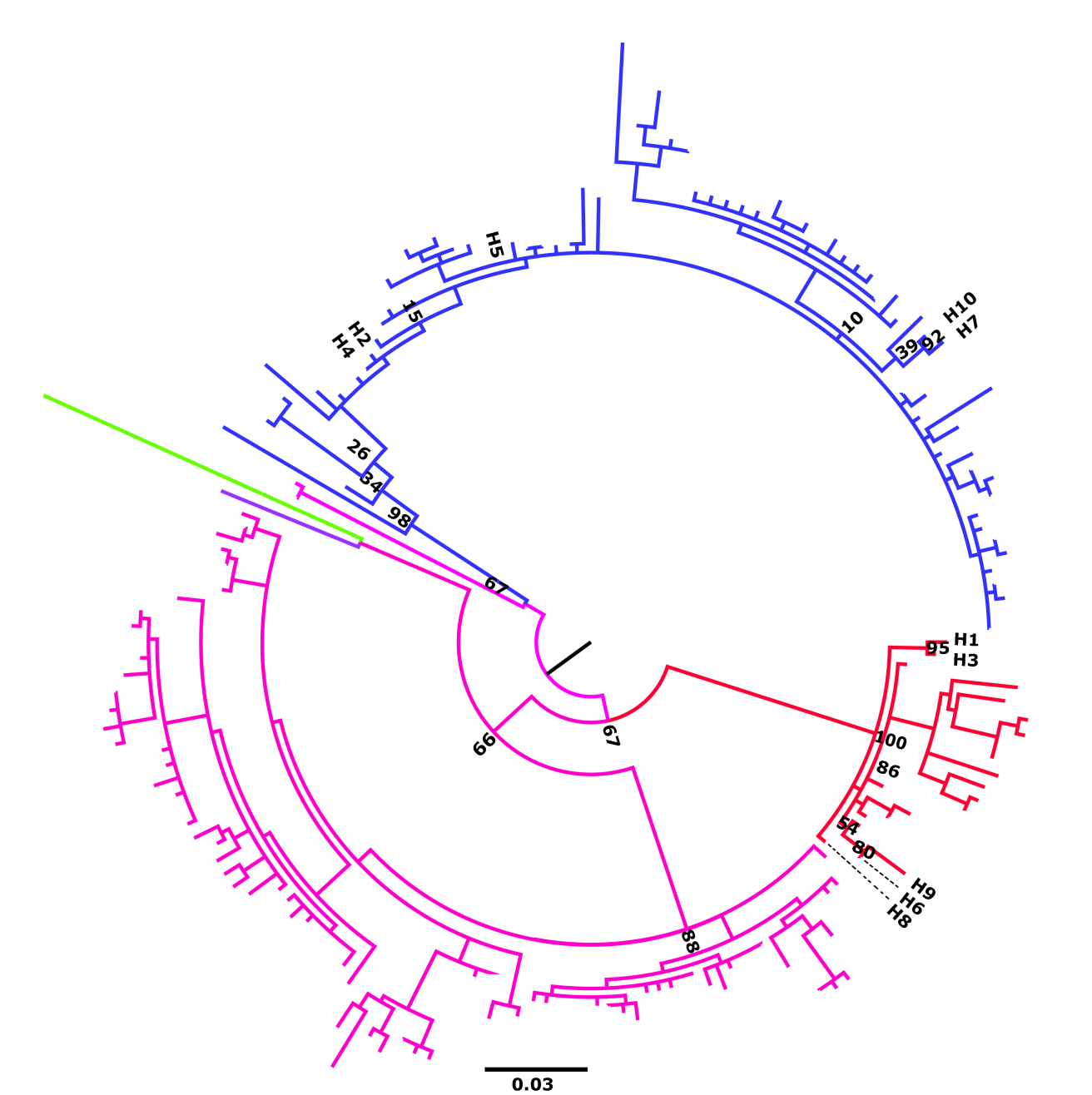


Figure S6: Unrooted phylogenetic tree of *hcpA*, obtained by ML. Alleles described in *C. parallelus* are named H1 to H10. Posterior probabilities are shown in the nodes. The colour code indicates supergroups A (pink), B (blue), D (green), F (red) and H (purple). Bootstrap values are shown in the nodes.

*
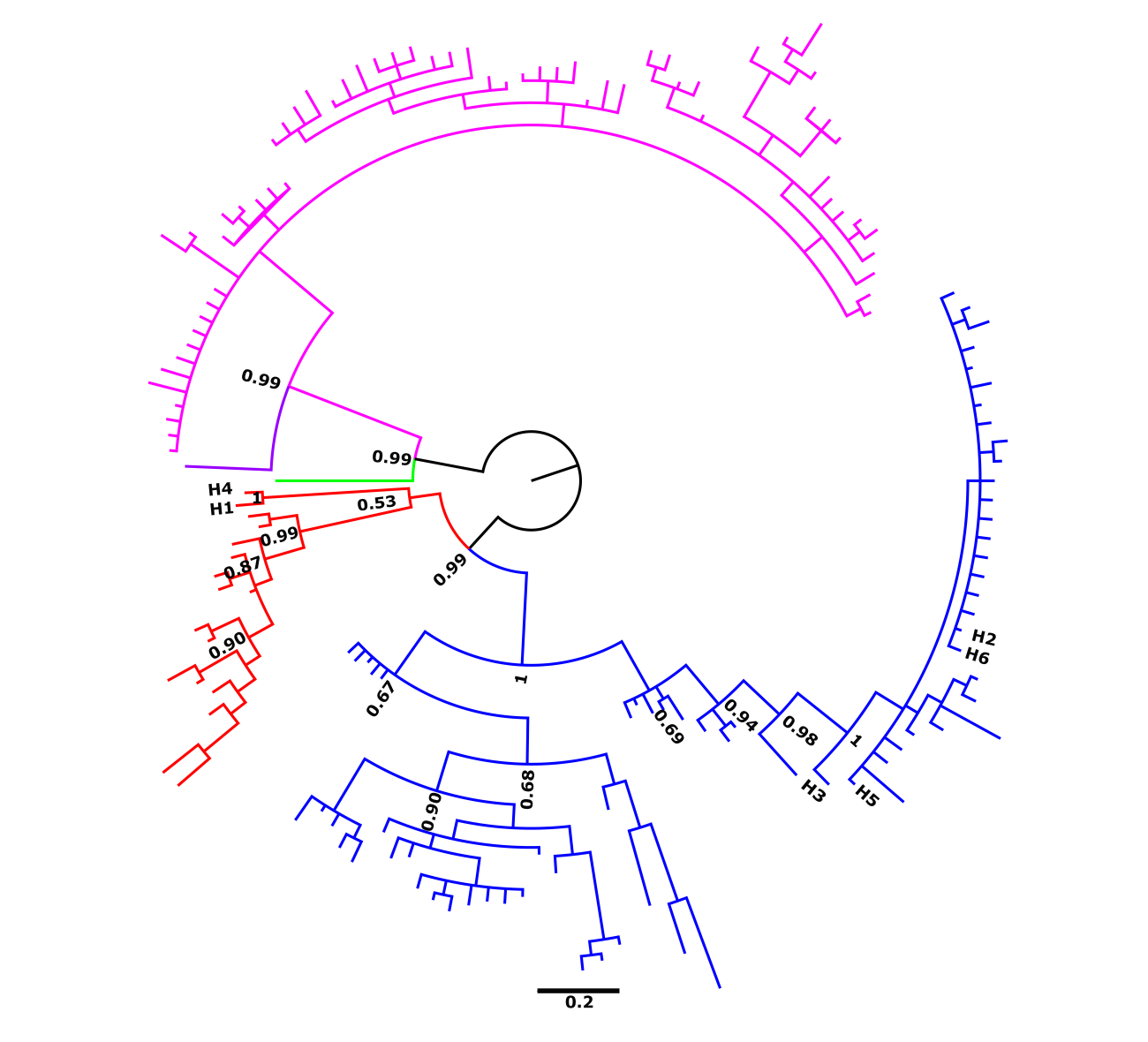
*

Figure S7: Unrooted phylogenetic tree of *coxA*, obtained by Bayesian inference. Alleles described in *C. parallelus* are named H1 to H6. Posterior probabilities are shown in the nodes. The colour code indicates supergroups A (pink), B (blue), D (green), F (red) and H (purple). Posterior probabilities are shown in the nodes.


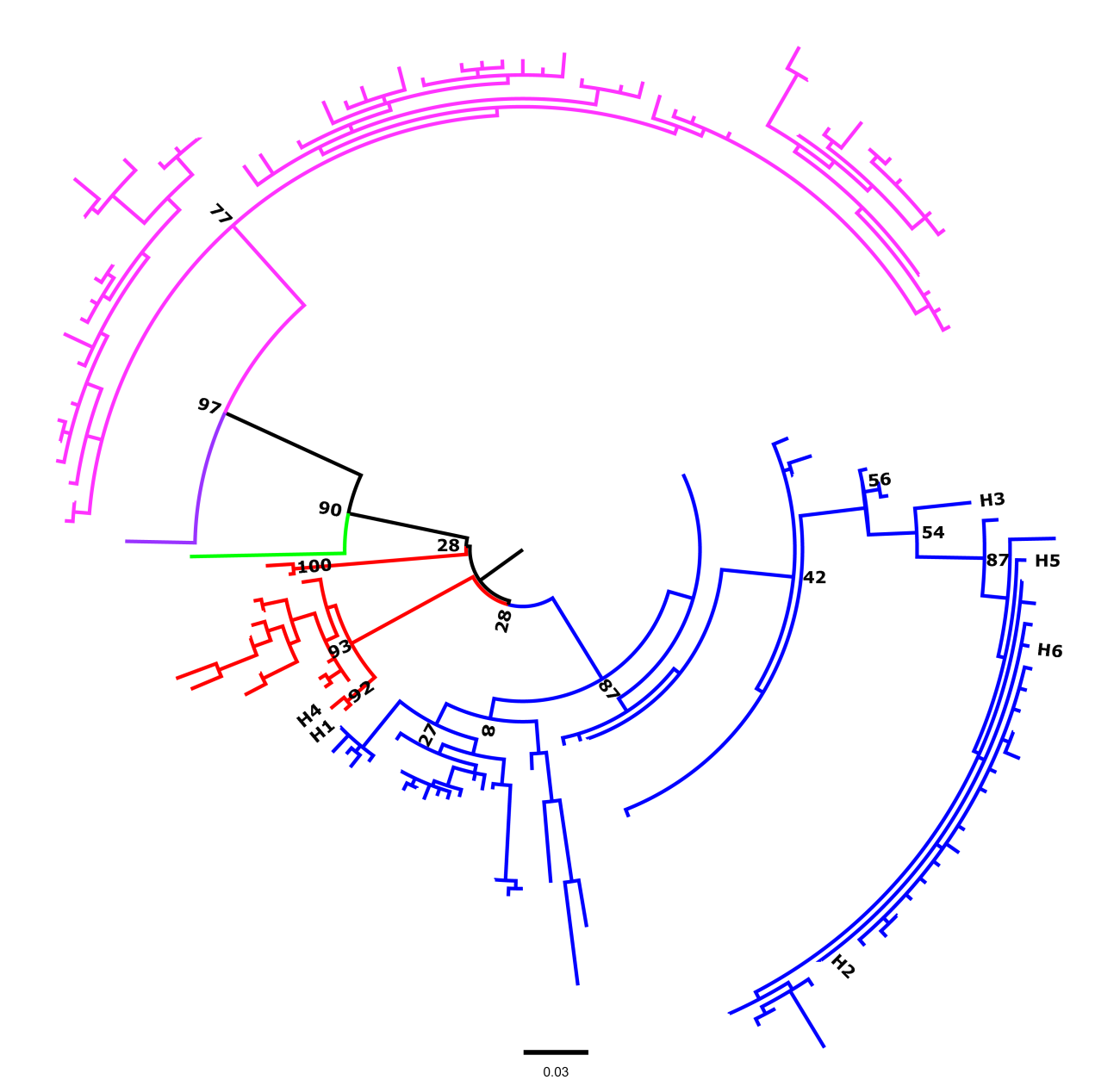


Figure S8: Unrooted phylogenetic tree of *coxA*, obtained by ML. Alleles described in *C. parallelus* are named H1 to H6. Posterior probabilities are shown in the nodes. The colour code indicates supergroups A (pink), B (blue), D (green), F (red) and H (purple). Bootstrap values are shown in the nodes.


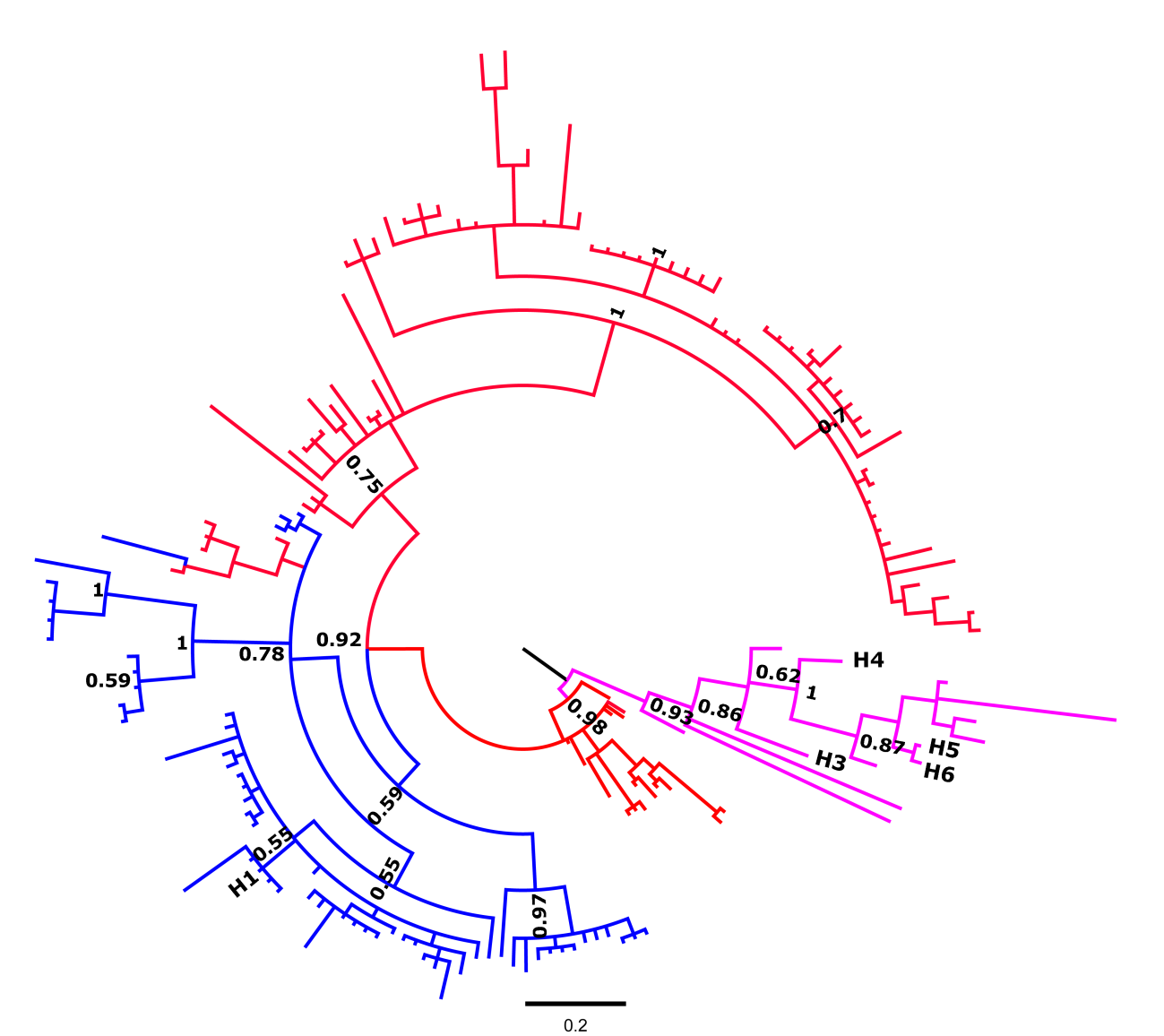


Figure S9: Unrooted phylogenetic tree of *wsp,* obtained by Bayesian inference. Alleles described in *C. parallelus* are named H1 to H16. Posterior probabilities are shown in the nodes. The colour code indicates supergroups A (pink), B (blue), D (green), F (red) and H (purple). Posterior probabilities are shown in the nodes.


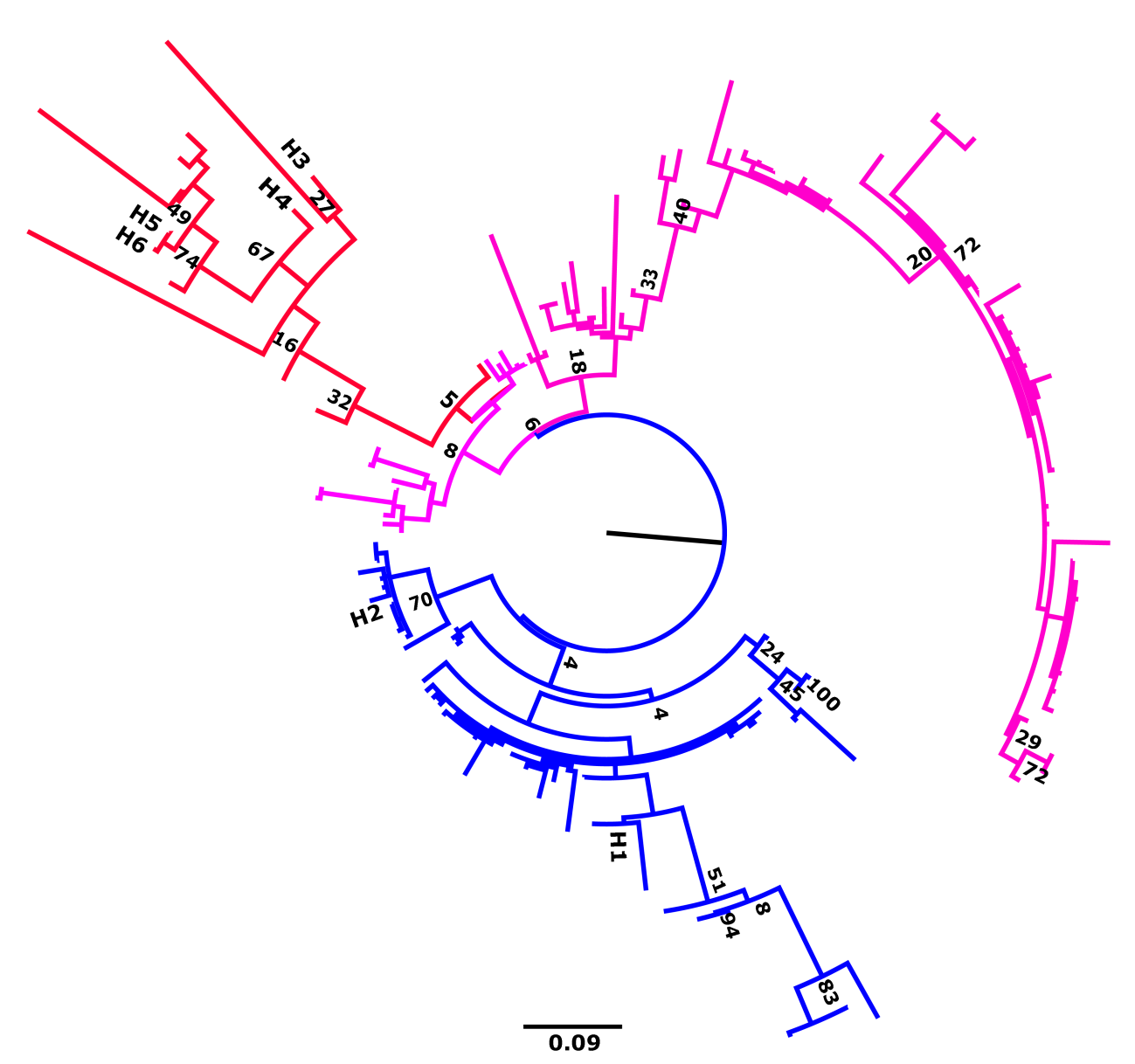


Figure S10: Unrooted phylogenetic tree of *wsp,* obtained by Bayesian inference. Alleles described in *C. parallelus* are named H1 to H16. Posterior probabilities are shown in the nodes. The colour code indicates supergroups A (pink), B (blue), D (green), F (red) and H (purple). Bootstrap values are shown in the nodes.

*
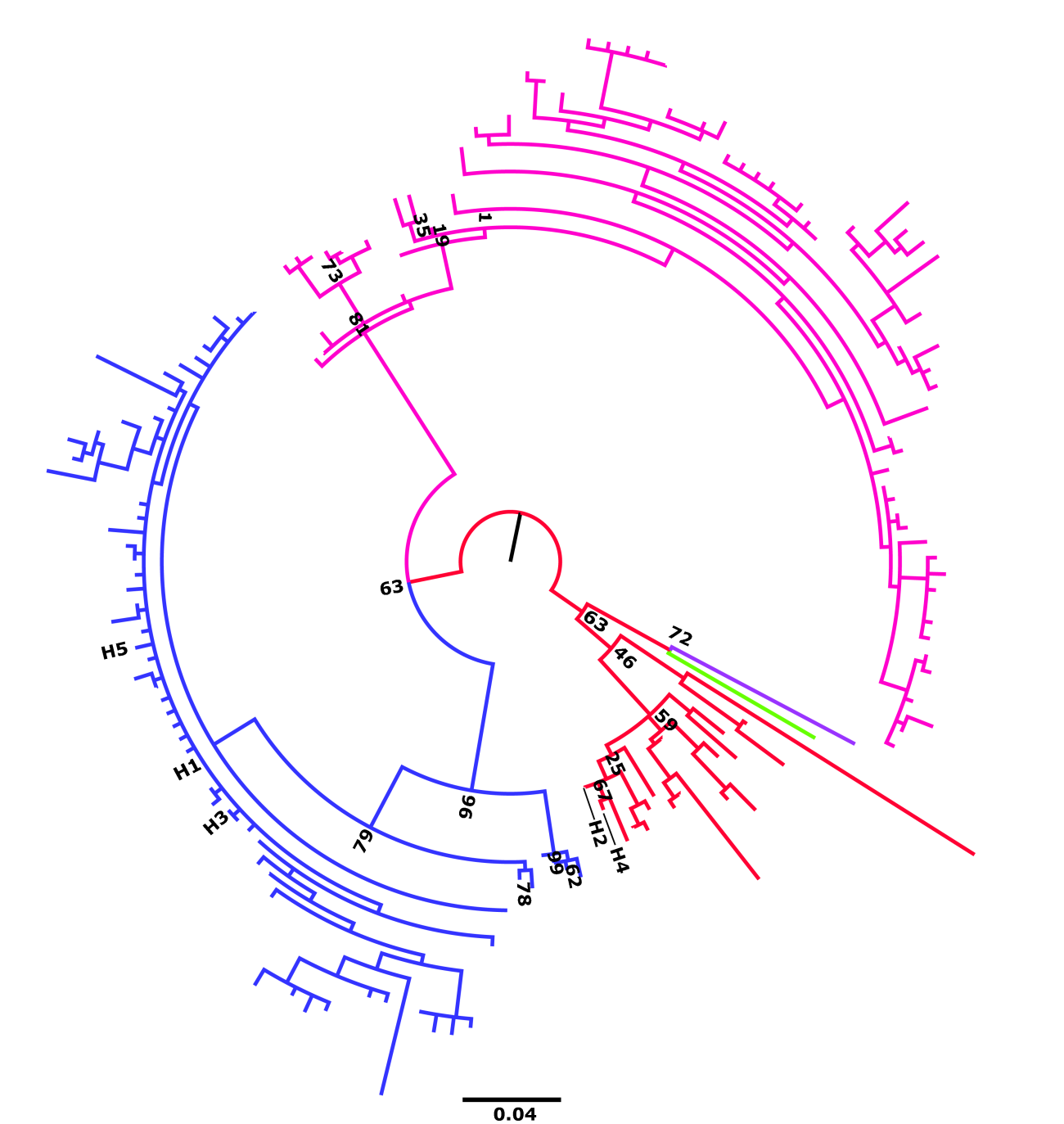
*

Figure S11: Unrooted phylogenetic tree of *GatB,* obtained by Bayesian inference. Alleles described in *C. parallelus* are named H1 to H16. Posterior probabilities are shown in the nodes. The colour code indicates supergroups A (pink), B (blue), D (green), F (red) and H (purple). Bootstrap values are shown in the nodes.


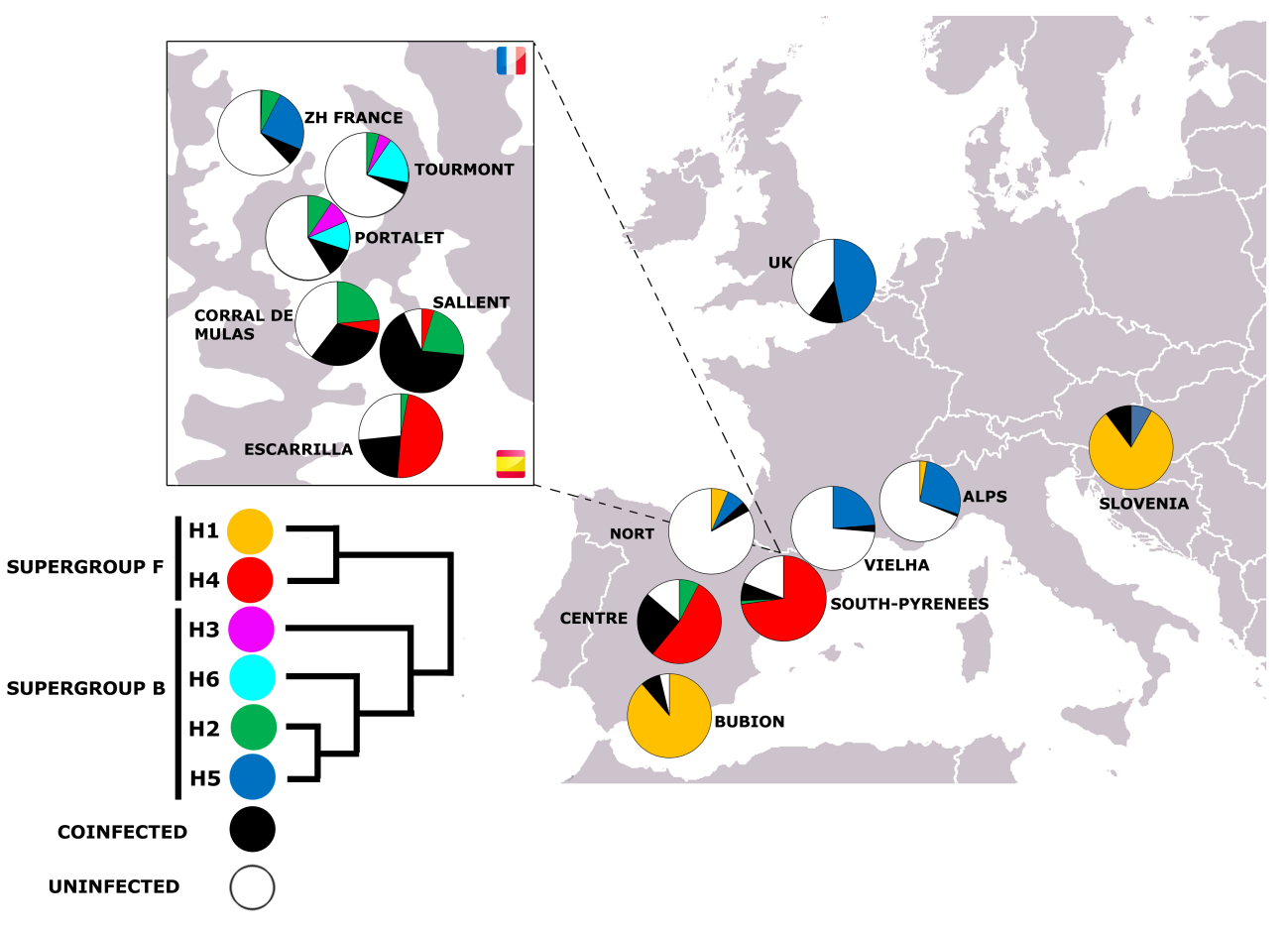


Figure S12: Geographical distribution of detected *coxA* alleles.


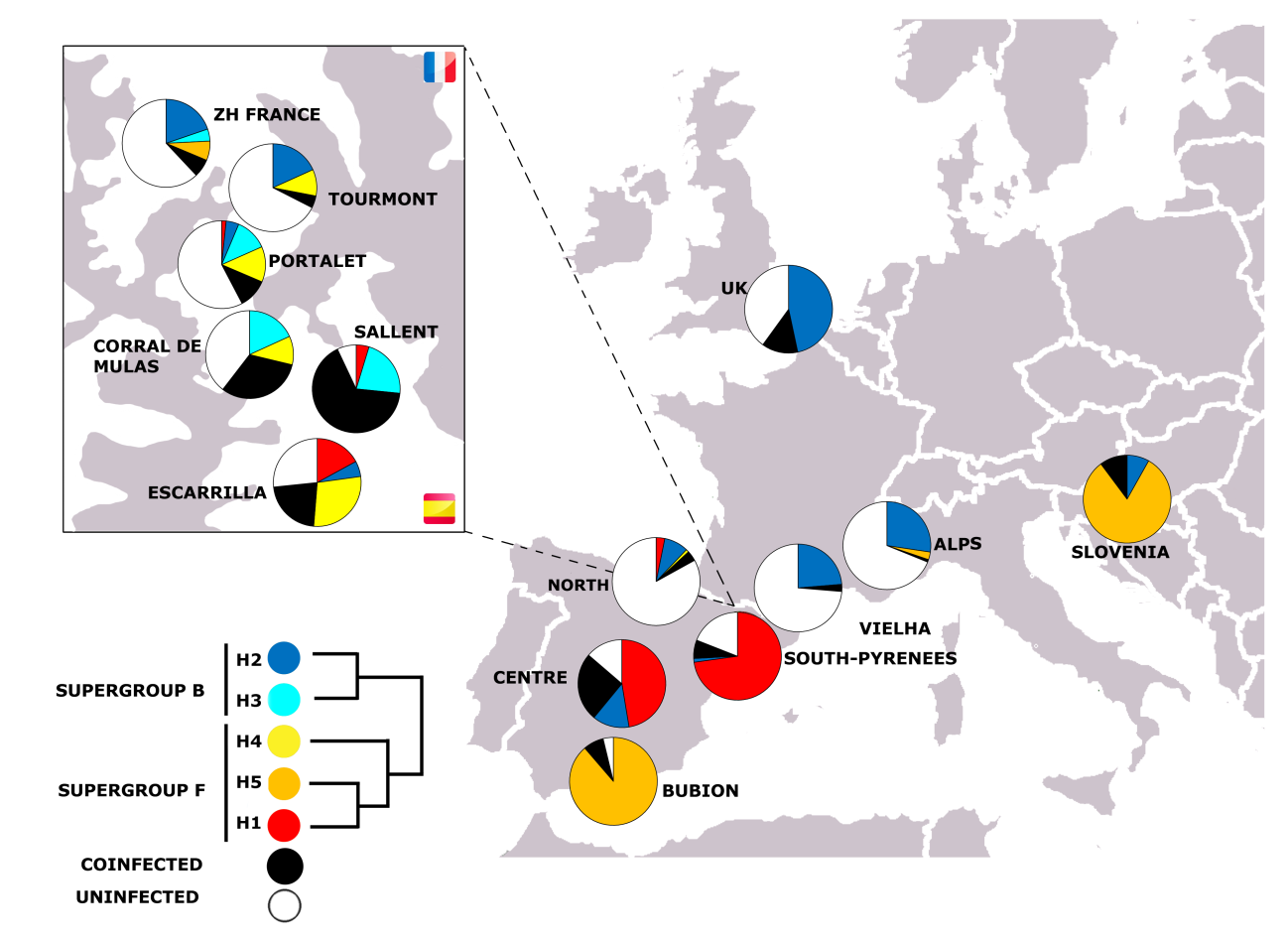


Figure S13: Geographical distribution of detected *fbpA* alleles.


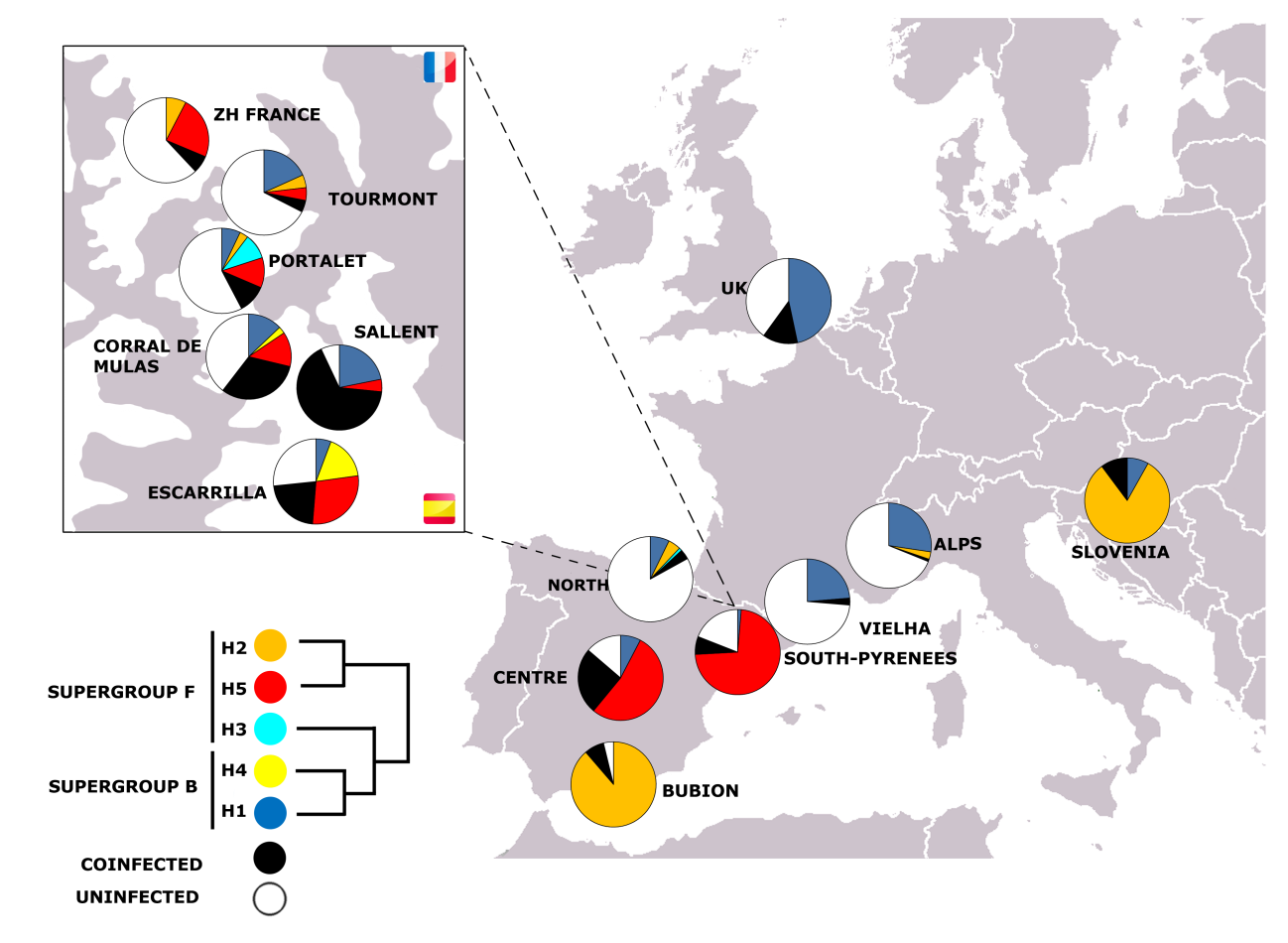


Figure S14: Geographical distribution of detected *ftsZ* alleles.


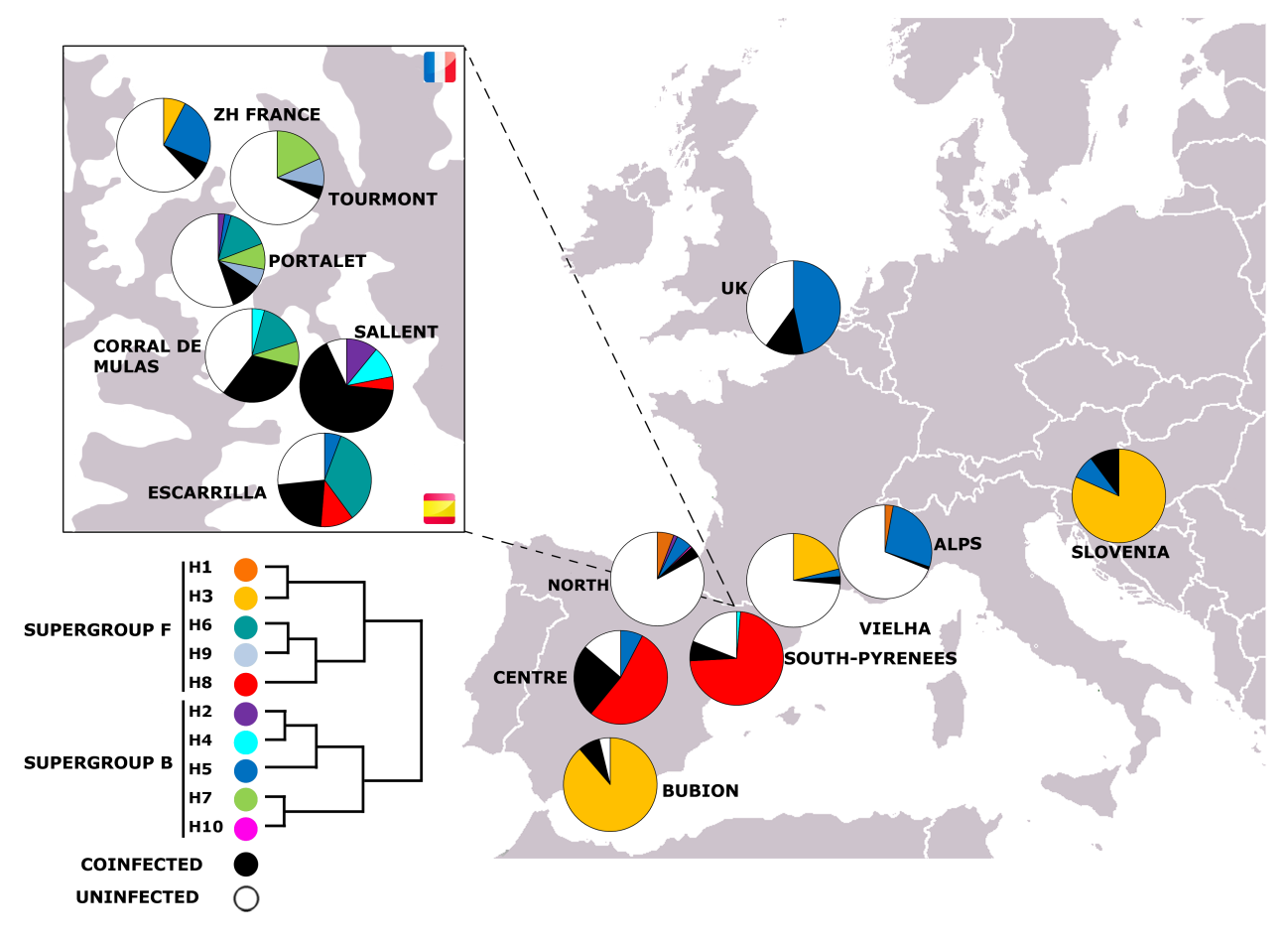


Figure S15: Geographical distribution of detected *hcpA* alleles.


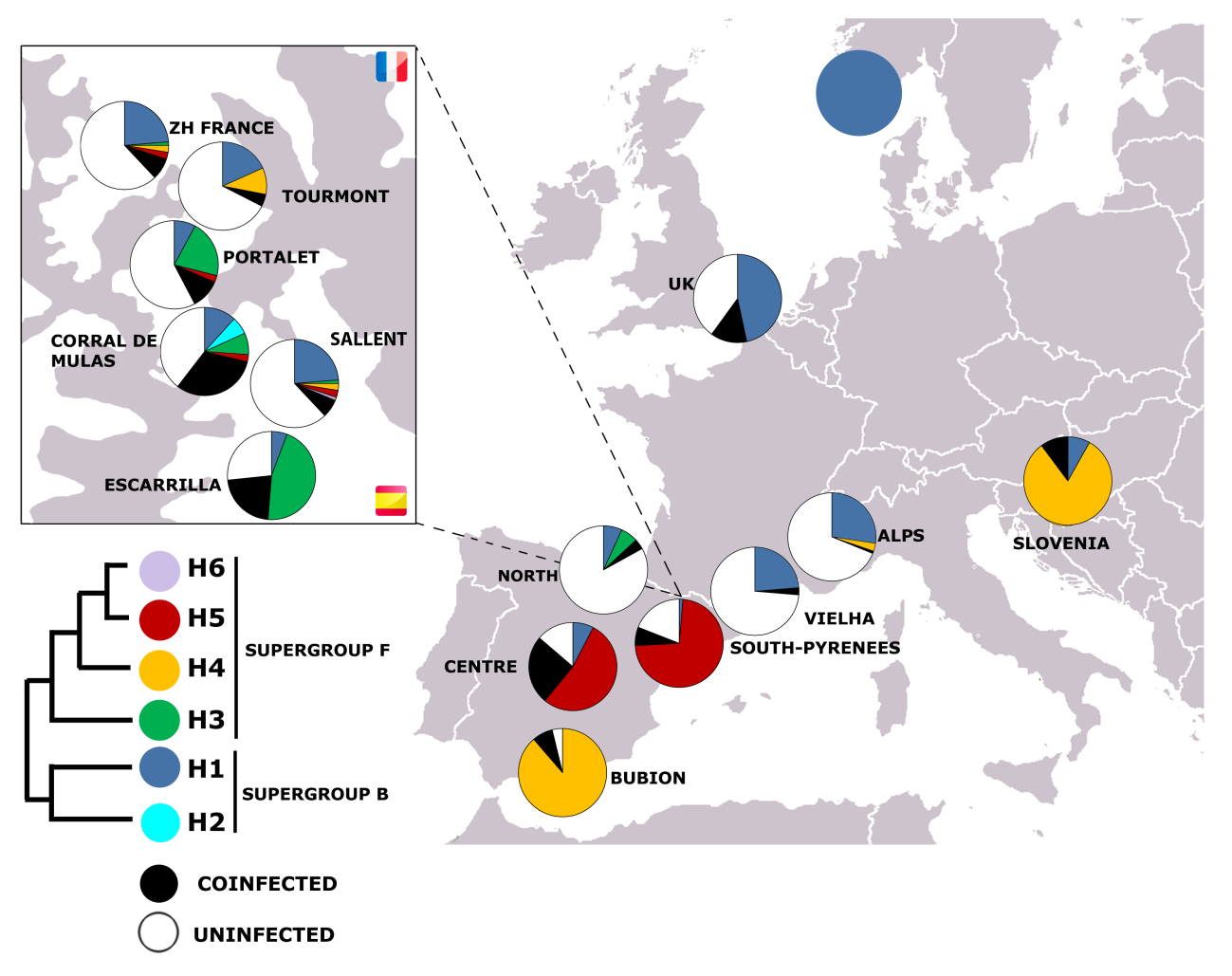


Figure S16: Geographical distribution of *wsp* alleles.


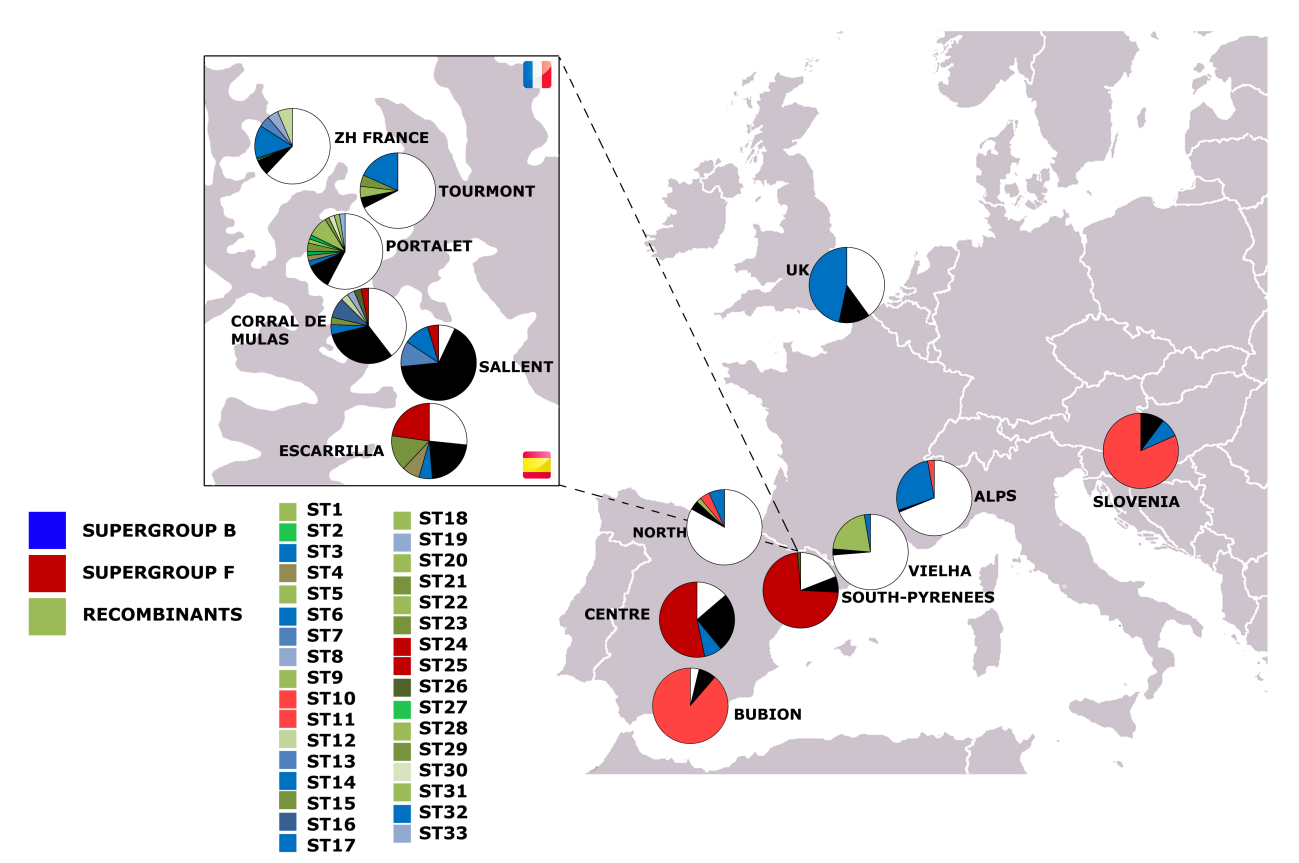


Figure S17: Geographical distribution of STs.

**Supplementary Tables:**

Table S1: Locus-by-locus AMOVA implemented in ARLEQUIN.

|  | Among groups | | | | Among populations, between groups | | | | Within population | | | | Fixation index | | | | | |
| --- | --- | --- | --- | --- | --- | --- | --- | --- | --- | --- | --- | --- | --- | --- | --- | --- | --- | --- |
| Locus | SSD | df | Va (%) | Variation | SSD | df | Vb (%) | Variation | SSD | df | Vc (%) | Variation | F_SC_ | P | F_ST_ | P | F_CT_ | P |
| *gatB* | 1437156 | 3 | 0*.*28 | 80*.*20 | 116632 | 9 | 0*.*02 | 5*.*86 | 258333 | 53 | 0*.*05 | 13*.*95 | 0*.*2957 | 0*.*0557 | 0*.*8605 | 0*.*0000 | 0*.*8020 | 0*.*0029 |
| *coxA* | 1809649 | 3 | 0*.*35 | 73*.*60 | 208533 | 9 | 0*.*04 | 7*.*74 | 465152 | 53 | 0*.*09 | 18*.*65 | 0*.*2934 | 0*.*0059 | 0*.*8135 | 0*.*0000 | 0*.*7360 | 0*.*0000 |
| *HcpA* | 1304275 | 3 | 0*.*17 | 39*.*07 | 756331 | 9 | 0*.*20 | 45*.*04 | 365152 | 53 | 0*.*07 | 15*.*89 | 0*.*7392 | 0*.*0000 | 0*.*8411 | 0*.*0000 | 0*.*3907 | 0*.*0166 |
| *ftsZ* | 1096416 | 3 | 0*.*20 | 53*.*22 | 270250 | 9 | 0*.*04 | 11*.*91 | 677273 | 53 | 0*.*13 | 34*.*87 | 0*.*2547 | 0*.*0274 | 0*.*6513 | 0*.*0000 | 0*.*5322 | 0*.*0039 |
| *fbpA* | 1280584 | 3 | 0*.*20 | 47*.*92 | 486841 | 9 | 0*.*11 | 25*.*65 | 593182 | 53 | 0*.*11 | 26*.*43 | 0*.*4925 | 0*.*0000 | 0*.*7357 | 0*.*0000 | 0*.*4792 | 0*.*0068 |

Table S2: Exact test of population differentiation following the methodology of [Rousset et al*.* (1992](#_ENREF_3)), i.e. test of non-random distribution of haplotypes into population samples under the hypothesis of panmixia, implemented in ARLEQUIN. Grey = pairs of populations between which differentiation was observed (statistically significant). ± = ARLEQUIN provided errors.

|  | Puerto Cantó | Centre | Escarrilla | Sallent | Alps | North | Vielha | Bubión | Slovenia | Tourmont | Portalet | Corral de Mulas | HZ (Fr) |
| --- | --- | --- | --- | --- | --- | --- | --- | --- | --- | --- | --- | --- | --- |
| Puerto Cantó |  |  |  |  |  |  |  |  |  |  |  |  |  |
| Centre | 0*.*00458 ± 0*.*0008 |  |  |  |  |  |  |  |  |  |  |  |  |
| Escarrilla | 0*.*10008 ± 0*.*0021 | 0*.*00216 ± 0*.*0005 |  |  |  |  |  |  |  |  |  |  |  |
| Sallent | 0*.*24378 ± 0*.*0048 | 0*.*00015 ± 0*.*0001 | 0*.*10440 ± 0*.*0042 |  |  |  |  |  |  |  |  |  |  |
| Alps | 0*.*06187 ± 0*.*0015 | 0*.*00021 ± 0*.*0001 | 0*.*03494 ± 0*.*0017 | 0*.*39706 ± 0*.*0041 |  |  |  |  |  |  |  |  |  |
| North | 0*.*10136 ± 0*.*0015 | 0*.*00207 ± 0*.*0004 | ≈ 0*.*00000 | 0*.*10483 ± 0*.*0028 | 0*.*03525 ± 0*.*0011 |  |  |  |  |  |  |  |  |
| Vielha | 0*.*01245 ± 0*.*0010 | 0*.*00002 ± 0*.*0000 | 0*.*01049 ± 0*.*0008 | 0*.*00210 ± 0*.*0006 | 0*.*00247 ± 0*.*0005 | 0*.*01230 ± 0*.*0006 |  |  |  |  |  |  |  |
| Bubión | 0*.*05564 ± 0*.*0030 | ≈ 0*.*00000 | 0*.*02453 ± 0*.*0024 | 0*.*03982 ± 0*.*0011 | 0*.*00212 ± 0*.*0004 | 0*.*02717 ± 0*.*0008 | 0*.*00013 ± 0*.*0001 |  |  |  |  |  |  |
| Slovenia | 0*.*50524 ± 0*.*0020 | 0*.*15809 ± 0*.*0027 | 0*.*25167 ± 0*.*0021 | 0*.*70584 ± 0*.*0059 | 0*.*27881 ± 0*.*0042 | 0*.*24826 ± 0*.*0023 | ≈ 0*.*00000 | 0*.*42498 ± 0*.*0123 |  |  |  |  |  |
| Tourmont | 0*.*01702 ± 0*.*0008 | 0*.*00038 ± 0*.*0002 | 0*.*01731 ± 0*.*0006 | 0*.*01048 ± 0*.*0013 | 0*.*00451 ± 0*.*0004 | 0*.*01802 ± 0*.*0009 | ≈ 0*.*00000 | 0*.*00146 ± 0*.*0004 | ≈ 0*.*00000 |  |  |  |  |
| Portalet | 0*.*00802 ± 0*.*0009 | ≈ 0*.*00000 | ≈ 0*.*00000 | 0*.*00049 ± 0*.*0003 | 0*.*00033 ± 0*.*0002 | ≈ 0*.*00000 | 0*.*00052 ± 0*.*0001 | 0*.*00046 ± 0*.*0003 | 0*.*12316 ± 0*.*0015 | 0*.*00135 ± 0*.*0003 |  |  |  |
| Corral de Mulas | 0*.*39703 ± 0*.*0045 | 0*.*03246 ± 0*.*0018 | 0*.*09899 ± 0*.*0013 | 0*.*57693 ± 0*.*0065 | 0*.*14184 ± 0*.*0023 | 0*.*10019 ± 0*.*0014 | 0*.*03558 ± 0*.*0014 | 0*.*37234 ± 0*.*0104 | 1*.*00000 ± 0*.*0000 | 0*.*04496 ± 0*.*0012 | 0*.*02705 ± 0*.*0015 |  |  |
| HZ France | 0*.*49858 ± 0*.*0032 | 0*.*15600 ± 0*.*0035 | 0*.*24915 ± 0*.*0022 | 0*.*70848 ± 0*.*0057 | 0*.*28748 ± 0*.*0067 | 0*.*25371 ± 0*.*0019 | 0*.*14421 ± 0*.*0017 | 0*.*41815 ± 0*.*0090 | 1*.*00000 ± 0*.*0000 | 0*.*16910 ± 0*.*0021 | 0*.*12423 ± 0*.*0018 | 1*.*00000 ± 0*.*0000 |  |

Table S3: Alleles included in the phylogenetic analysis of gatB (see <https://pubmlst.org/wolbachia/>).

| gatB_1 | gatB_21 | gatB_121 | gatB_31 | gatB_97 | gatB_23 | gatB_66 |
| --- | --- | --- | --- | --- | --- | --- |
| gatB_2 | gatB_38 | gatB_122 | gatB_30 | gatB_129 | gatB_36 | gatB_72 |
| gatB_62 | gatB_39 | gatB_126 | gatB_73 | gatB_131 | gatB_32 | gatB_128 |
| gatB_68 | gatB_40 | gatB_127 | gatB_65 | gatB_141 | gatB_49 | gatB_99 |
| gatB_88 | gatB_48 | gatB_132 | gatB_82 | gatB_142 | gatB_53 | gatB_130 |
| gatB_89 | gatB_55 | gatB_134 | gatB_113 | gatB_143 | gatB_56 | gatB_138 |
| gatB_3 | gatB_69 | gatB_139 | gatB_112 | gatB_18 | gatB_76 | gatB_157 |
| gatB_4 | gatB_70 | gatB_140 | gatB_115 | gatB_46 | gatB_94 | gatB_22 |
| gatB_83 | gatB_71 | gatB_145 | gatB_116 | gatB_133 | gatB_98 |  |
| gatB_101 | gatB_125 | gatB_147 | gatB_117 | gatB_90 | gatB_123 |  |
| gatB_135 | gatB_79 | gatB_149 | gatB_118 | gatB_33 | gatB_37 |  |
| gatB_137 | gatB_80 | gatB_150 | gatB_81 | gatB_34 | gatB_78 |  |
| gatB_152 | gatB_91 | gatB_151 | gatB_110 | gatB_124 | gatB_93 |  |
| gatB_96 | gatB_100 | gatB_153 | gatB_111 | gatB_19 | gatB_42 |  |
| gatB_148 | gatB_102 | gatB_155 | gatB_28 | gatB_20 | gatB_43 |  |
| gatB_5 | gatB_154 | gatB_158 | gatB_64 | gatB_24 | gatB_75 |  |
| gatB_6 | gatB_103 | gatB_15 | gatB_7 | gatB_27 | gatB_45 |  |
| gatB_17 | gatB_104 | gatB_156 | gatB_47 | gatB_41 | gatB_54 |  |
| gatB_25 | gatB_105 | gatB_59 | gatB_67 | gatB_74 | gatB_87 |  |
| gatB_136 | gatB_106 | gatB_95 | gatB_85 | gatB_35 | gatB_57 |  |
| gatB_108 | gatB_107 | gatB_144 | gatB_86 | gatB_51 | gatB_58 |  |
| gatB_9 | gatB_146 | gatB_92 | gatB_8 | gatB_52 | gatB_60 |  |
| gatB_12 | gatB_109 | gatB_26 | gatB_10 | gatB_44 | gatB_61 |  |
| gatB_13 | gatB_119 | gatB_114 | gatB_11 | gatB_50 | gatB_63 |  |
| gatB_16 | gatB_120 | gatB_29 | gatB_14 | gatB_77 | gatB_84 |  |

Table S4: Alleles included in the phylogenetic analysis of coxA (see <https://pubmlst.org/wolbachia/>).

| coxA_1 | coxA_105 | coxA_131 | coxA_87 | coxA_129 | coxA_32 |
| --- | --- | --- | --- | --- | --- |
| coxA_6 | coxA_106 | coxA_83 | coxA_91 | coxA_2 | coxA_61 |
| coxA_17 | coxA_122 | coxA_13 | coxA_112 | coxA_60 | coxA_10 |
| coxA_23 | coxA_123 | coxA_5 | coxA_27 | coxA_70 | coxA_37 |
| coxA_103 | coxA_126 | coxA_88 | coxA_69 | coxA_110 | coxA_58 |
| coxA_111 | coxA_133 | coxA_96 | coxA_94 | coxA_20 | coxA_130 |
| coxA_113 | coxA_139 | coxA_25 | coxA_82 | coxA_24 | coxA_84 |
| coxA_116 | coxA_9 | coxA_11 | coxA_95 | coxA_28 | coxA_50 |
| coxA_117 | coxA_80 | coxA_38 | coxA_97 | coxA_35 | coxA_53 |
| coxA_118 | coxA_22 | coxA_79 | coxA_92 | coxA_45 | coxA_77 |
| coxA_124 | coxA_78 | coxA_121 | coxA_93 | coxA_46 | coxA_44 |
| coxA_143 | coxA_68 | coxA_135 | coxA_30 | coxA_40 | coxA_48 |
| coxA_140 | coxA_16 | coxA_136 | coxA_31 | coxA_41 | coxA_49 |
| coxA_141 | coxA_119 | coxA_144 | coxA_55 | coxA_21 | coxA_52 |
| coxA_3 | coxA_137 | coxA_138 | coxA_56 | coxA_108 | coxA_59 |
| coxA_8 | coxA_64 | coxA_43 | coxA_63 | coxA_109 | coxA_72 |
| coxA_114 | coxA_73 | coxA_67 | coxA_89 | coxA_33 | coxA_104 |
| coxA_12 | coxA_132 | coxA_120 | coxA_90 | coxA_34 | coxA_57 |
| coxA_14 | coxA_134 | coxA_98 | coxA_29 | coxA_39 | coxA_62 |
| coxA_18 | coxA_26 | coxA_99 | coxA_54 | coxA_74 |  |
| coxA_36 | coxA_107 | coxA_100 | coxA_15 | coxA_47 |  |
| coxA_51 | coxA_65 | coxA_101 | coxA_42 | coxA_75 |  |
| coxA_81 | coxA_66 | coxA_102 | coxA_76 | coxA_142 |  |
| coxA_115 | coxA_125 | coxA_71 | coxA_127 | coxA_7 |  |
| coxA_85 | coxA_4 | coxA_86 | coxA_128 | coxA_19 |  |

Table S5: Alleles included in the phylogenetic analysis of fbpA (see <https://pubmlst.org/wolbachia/>).

| fbpA_1 | fbpA_192 | fbpA_112 | fbpA_147 |
| --- | --- | --- | --- |
| fbpA_2 | fbpA_205 | fbpA_98 | fbpA_186 |
| fbpA_92 | fbpA_150 | fbpA_113 | fbpA_199 |
| fbpA_10 | fbpA_157 | fbpA_116 | fbpA_220 |
| fbpA_21 | fbpA_16 | fbpA_117 | fbpA_121 |
| fbpA_142 | fbpA_195 | fbpA_123 |  |
| fbpA_6 | fbpA_201 | fbpA_65 |  |
| fbpA_91 | fbpA_219 | fbpA_15 |  |
| fbpA_137 | fbpA_41 | fbpA_58 |  |
| fbpA_181 | fbpA_125 | fbpA_66 |  |
| fbpA_185 | fbpA_129 | fbpA_79 |  |
| fbpA_141 | fbpA_145 | fbpA_61 |  |
| fbpA_143 | fbpA_169 | fbpA_154 |  |
| fbpA_155 | fbpA_70 | fbpA_163 |  |
| fbpA_214 | fbpA_73 | fbpA_179 |  |
| fbpA_4 | fbpA_57 | fbpA_19 |  |
| fbpA_43 | fbpA_101 | fbpA_36 |  |
| fbpA_75 | fbpA_103 | fbpA_47 |  |
| fbpA_76 | fbpA_106 | fbpA_52 |  |
| fbpA_176 | fbpA_107 | fbpA_54 |  |
| fbpA_25 | fbpA_50 | fbpA_85 |  |
| fbpA_95 | fbpA_140 | fbpA_96 |  |
| fbpA_162 | fbpA_86 | fbpA_8 |  |
| fbpA_207 | fbpA_108 | fbpA_83 |  |
| fbpA_132 | fbpA_110 | fbpA_119 |  |

Table S6: Alleles included in the phylogenetic analysis of *ftsZ* (see <https://pubmlst.org/wolbachia/>).

| ftsZ_1 | ftsZ_98 | ftsZ_22 | ftsZ_66 | ftsZ_33 |
| --- | --- | --- | --- | --- |
| ftsZ_3 | ftsZ_99 | ftsZ_106 | ftsZ_63 | ftsZ_58 |
| ftsZ_5 | ftsZ_100 | ftsZ_108 | ftsZ_36 | ftsZ_14 |
| ftsZ_6 | ftsZ_103 | ftsZ_110 | ftsZ_41 | ftsZ_50 |
| ftsZ_10 | ftsZ_104 | ftsZ_109 | ftsZ_65 | ftsZ_13 |
| ftsZ_17 | ftsZ_116 | ftsZ_111 | ftsZ_73 | ftsZ_56 |
| ftsZ_29 | ftsZ_51 | ftsZ_4 | ftsZ_80 | ftsZ_74 |
| ftsZ_32 | ftsZ_53 | ftsZ_7 | ftsZ_78 | ftsZ_113 |
| ftsZ_34 | ftsZ_54 | ftsZ_8 | ftsZ_81 | ftsZ_114 |
| ftsZ_38 | ftsZ_24 | ftsZ_77 | ftsZ_89 | ftsZ_115 |
| ftsZ_39 | ftsZ_85 | ftsZ_107 | ftsZ_90 | ftsZ_79 |
| ftsZ_40 | ftsZ_27 | ftsZ_9 | ftsZ_94 | ftsZ_92 |
| ftsZ_42 | ftsZ_59 | ftsZ_11 | ftsZ_97 | ftsZ_93 |
| ftsZ_43 | ftsZ_28 | ftsZ_12 | ftsZ_95 | ftsZ_30 |
| ftsZ_44 | ftsZ_48 | ftsZ_96 | ftsZ_101 | ftsZ_31 |
| ftsZ_45 | ftsZ_60 | ftsZ_15 | ftsZ_105 | ftsZ_75 |
| ftsZ_47 | ftsZ_84 | ftsZ_62 | ftsZ_117 | ftsZ_76 |
| ftsZ_49 | ftsZ_68 | ftsZ_102 | ftsZ_19 |  |
| ftsZ_52 | ftsZ_61 | ftsZ_112 | ftsZ_91 |  |
| ftsZ_55 | ftsZ_86 | ftsZ_69 | ftsZ_16 |  |
| ftsZ_57 | ftsZ_88 | ftsZ_35 | ftsZ_21 |  |
| ftsZ_64 | ftsZ_87 | ftsZ_71 | ftsZ_67 |  |
| ftsZ_70 | ftsZ_26 | ftsZ_18 | ftsZ_37 |  |
| ftsZ_72 | ftsZ_83 | ftsZ_20 | ftsZ_25 |  |
| ftsZ_82 | ftsZ_2 | ftsZ_23 | ftsZ_46 |  |

Table S7: Alleles included in the phylogenetic analysis of *hcpA* (see <https://pubmlst.org/wolbachia/>).

| hcpA_106 | hcpA_49 | hcpA_69 | hcpA_76 | hcpA_114 | hcpA_119 | hcpA_153 |
| --- | --- | --- | --- | --- | --- | --- |
| hcpA_130 | hcpA_46 | hcpA_96 | hcpA_134 | hcpA_115 | hcpA_164 | hcpA_158 |
| hcpA_144 | hcpA_54 | hcpA_61 | hcpA_18 | hcpA_116 | hcpA_136 | hcpA_157 |
| hcpA_13 | hcpA_45 | hcpA_64 | hcpA_131 | hcpA_112 | hcpA_151 | hcpA_160 |
| hcpA_135 | hcpA_78 | hcpA_24 | hcpA_95 | hcpA_73 | hcpA_152 | hcpA_156 |
| hcpA_12 | hcpA_28 | hcpA_63 | hcpA_60 | hcpA_117 | hcpA_146 | hcpA_159 |
| hcpA_150 | hcpA_133 | hcpA_59 | hcpA_16 | hcpA_107 | hcpA_102 | hcpA_140 |
| hcpA_166 | hcpA_97 | hcpA_92 | hcpA_23 | hcpA_108 | hcpA_99 | hcpA_155 |
| hcpA_165 | hcpA_75 | hcpA_149 | hcpA_83 | hcpA_14 | hcpA_154 | hcpA_74 |
| hcpA_137 | hcpA_26 | hcpA_37 | hcpA_47 | hcpA_124 | hcpA_161 | hcpA_5 |
| hcpA_138 | hcpA_86 | hcpA_65 | hcpA_82 | hcpA_121 | hcpA_9 | hcpA_4 |
| hcpA_1 | hcpA_167 | hcpA_11 | hcpA_127 | hcpA_120 | hcpA_91 | hcpA_15 |
| hcpA_27 | hcpA_2 | hcpA_128 | hcpA_21 | hcpA_122 | hcpA_104 | hcpA_10 |
| hcpA_68 | hcpA_51 | hcpA_62 | hcpA_71 | hcpA_101 | hcpA_148 | hcpA_142 |
| hcpA_103 | hcpA_98 | hcpA_89 | hcpA_33 | hcpA_147 | hcpA_145 | hcpA_141 |
| hcpA_8 | hcpA_39 | hcpA_90 | hcpA_36 | hcpA_168 | hcpA_52 | hcpA_30 |
| hcpA_43 | hcpA_38 | hcpA_41 | hcpA_34 | hcpA_29 | hcpA_126 | hcpA_113 |
| hcpA_32 | hcpA_42 | hcpA_93 | hcpA_58 | hcpA_20 | hcpA_25 | hcpA_87 |
| hcpA_56 | hcpA_94 | hcpA_84 | hcpA_35 | hcpA_132 | hcpA_100 |  |
| hcpA_57 | hcpA_81 | hcpA_50 | hcpA_72 | hcpA_88 | hcpA_125 |  |
| hcpA_55 | hcpA_105 | hcpA_85 | hcpA_77 | hcpA_109 | hcpA_6 |  |
| hcpA_48 | hcpA_53 | hcpA_67 | hcpA_110 | hcpA_3 | hcpA_17 |  |
| hcpA_129 | hcpA_44 | hcpA_70 | hcpA_111 | hcpA_163 | hcpA_143 |  |
| hcpA_79 | hcpA_123 | hcpA_7 | hcpA_31 | hcpA_19 | hcpA_139 |  |
| hcpA_22 | hcpA_66 | hcpA_80 | hcpA_118 | hcpA_40 | hcpA_162 |  |

Table S8: Alleles included in the phylogenetic analysis of *wsp* (see <https://pubmlst.org/wolbachia/>).

| wsp_111 | wsp_6 | wsp_49 |
| --- | --- | --- |
| wsp_31 | wsp_10 | wsp_40 |
| wsp_127 | wsp_26 | wsp_115 |
| wsp_5 | wsp_25 | wsp_117 |
| wsp_128 | wsp_7 | wsp_118 |
| wsp_9 | wsp_35 | wsp_120 |
| wsp_11 | wsp_36 | wsp_119 |
| wsp_22 | wsp_37 | wsp_122 |
| wsp_130 | wsp_74 | wsp_124 |
| wsp_14 | wsp_75 | wsp_151 |
| wsp_4 | wsp_76 | wsp_154 |
| wsp_1 | wsp_77 | wsp_157 |
| wsp_18 | wsp_79 | wsp_158 |
| wsp_21 | wsp_80 | wsp_101 |
| wsp_23 | wsp_113 | wsp_43 |
| wsp_33 | wsp_38 | wsp_41 |
| wsp_8 | wsp_87 | wsp_39 |
| wsp_29 | wsp_85 | wsp_45 |
| wsp_27 | wsp_90 | wsp_44 |
| wsp_28 | wsp_84 | wsp_83 |
| wsp_2 | wsp_103 |  |
| wsp_3 | wsp_89 |  |
| wsp_20 | wsp_106 |  |
| wsp_30 | wsp_46 |  |
| wsp_15 | wsp_48 |  |

# **Supplementary References**

Akaike, H. (1974). A new look at the statistical model identification. *IEEE Trans. Autom. Control* 19, 716–772.

Baldo, L., Bordenstein, S., Wernegreen, J.J. and Werren, J.H. (2006a) Widespread recombination throughout *Wolbachia* genomes. *Mol. Biol. Evol* 23, 437-449.

Baldo, L., Hotopp, J.C.D., Jolley, K.A., Bordenstein, S.R., Biber, S.A., Choudhury, R.R., Hayashi, C., Maiden, M.C.J., Tettelin, H. and Werren, J.H. (2006b). Multilocus sequence typing system for the endosymbiont *Wolbachia pipientis*. *App. Environ. Microbiol*. 72, 7098-7110.

Bella, J.L., Martínez-Rodríguez, P., Arroyo-Yebras, F., Bernal, A., Sarasa, J., Fernández-Calvín, B., Mason, P.L. and Zabal-Aguirre, M. (2010). *Wolbachia* infection in the *Chorthippus parallelus* hybrid zone: evidence for its role as a reproductive barrier*. J. Orthopt. Res*. 19, 205-212.

Didelot, X. and Falush, D. (2007. Inference of bacterial microevolution using multilocus sequence data. *Genetics* 175, 1251-1266.

Excoffier, L., Laval, G. and Schneider, S. (2005). ARLEQUIN ver. 3.0: an integrated software package for population genetics data analysis. *Evol. Bioinf. Online* 1, 47–50.

**Funkhouser-Jones, L.J., Sehnert, S.R., Martínez-Rodríguez, P., Toribio-Fernández, R., Pita, M., Bella, J.L. and Bordenstein, S.R. (2015). *Wolbachia* co-infection in a hybrid zone: discovery of horizontal gene transfers from two *Wolbachia* supergroups into an animal genome. *PeerJ*, 3: e1479.**

Jolley, K.A., Feil, E.J., Chan, M-S. and Maiden, M.C.J. (2001). Sequence type analysis and recombinational tests (START). *Bioinformatics* 17, 1230-1231.

Kishino, H. and Hasegawa, M. (1989). Evaluation of the maximum likelihood estimate of the evolutionary tree topologies from DNA sequence data, and the branching order in Hominoidea. *J. Mol. Evol.*, 29, 170-179.

Librado, P. and Rozas, J. (2009). DnaSP v5: A software for comprehensive analysis of DNA polymorphism data. *Bioinformatics* 25, 1451-1452.

Maiden, M.C.J., Bygraves, J.A., Feil, E, Morelli, G., Russell, J.E., Urwin, R., Zhang, Q., Zhou, J., Zurth, K., Caugant, D.A., Feavers, I.M., Achtman, M. and Spratt BG. (1998). Multilocus sequence typing: A portable approach to the identification of clones within populations of pathogenic microorganisms. *PNAS* 95, 3140-3145.

Martin, G., Delaunay, C., Braquart-Varnier, C. and Azzouna, A. (2010). Prophage elements from the endosymbiont, *Wolbachia* Hertig, 1936 transferred to the host genome of the woodlouse, *Armadillidium Vulgare* Latreille, 1804 (Peracarida, Isopoda). *Crustaceana* 83, 539-548.

Martínez-Rodríguez, P., Hernández-Pérez, M. and Bella J.L. (2013a). Detection of *Spiroplasma* and *Wolbachia* in the bacterial gonad community of *Chorthippus parallelus*. *Microb. Ecol.* 66, 211-223.

Martínez-Rodríguez, P., Sarasa, J., Peco, B, Jauregui, B.M., Rivera, D. and Bella, J.L. (2013b). Endosymbiont-free ants: molecular biological evidence that neither *Wolbachia*, *Cardinium* or any other bacterial endosymbionts play a role in thelytokous parthenogenesis in the Harvester ant species, *Messor barbarus* and *M. capitatus*. *Eur. J. Entomol.* 110, 197-204.

Maynard Smith, J. (1992). Analyzing the mosaic structure of genes. *J. Mol. Evol.* 34, 126-129.

Padidam, M., Sawyer, S. and Fauquet, C. (1999). Possible emergence of new geminiviruses by frequent recombination. *Virology* 265, 218-225.

Posada, D. (2008). jModelTest: Phylogenetic model averaging. *Mol. Biol. Evol.* 25, 1253-1256.

Posada, D. and Crandall, K. (2001). Evaluation of methods for detecting recombination from DNA sequences: Computer simulations. *PNAS* 98, 13757-13762.

Ronquist, F. and Huelsenbeck, J.P. (2003). MrBayes 3: Bayesian phylogenetic inference under mixed models. *Bioinformatics* 19, 1572-1574.

Rousset, F., Vautrin, D. and Solinag, M. (1992). Molecular-identification of *Wolbachia*, the agent of cytoplasmic incompatibility in *Drosophila* simulans, and variability in relation with host mitochondrial types *Proc. Royal Soc. London Series B-Biol. Sci.* 247, 163-168.

Schmidt, H.A., Strimmer, K., Vingron, M. and von Haeseler, A. (2002). TREE-PUZZLE: maximum likelihood phylogenetic analysis using quartets and parallel computing. *Bioinformatics* 18, 502-504.

Shimodaira, H. (2002). An approximately unbiased test of phylogenetic tree selection. *Systematic Biol.* 51, 492-508.

Shimodaira, H., Hasegawa, M. (1999). Multiple comparisons of log-likelihoods with applications to phylogenetic inference. *Mol. Biol. Evol.* 16, 1114.

Shimodaira, H. and Hasegawa, M. (2001). CONSEL: for assessing the confidence of phylogenetic tree selection. *Bioinformatics* 17, 1246-1247.

Stamatakis, A. (2006). RAxML-VI-HPC: Maximum likelihood-based phylogenetic analyses with thousands of taxa and mixed models. *Bioinformatics* 22, 2688-2690.

Stamatakis, A., Blagojevic, F., Nikolopoulos, D.S., Antonopoulos, C.D. (2007). Exploring new search algorithms and hardware for phylogenetics: RAxML meets the IBM cell. *J. VLSI Signal Process. Syst.* 48, 271-286.

**Toribio-Fernández, R., Bella, J.L., Martínez-Rodríguez, P., Funkhouser-Jones, L.J., Bordenstein, S.R. and Pita, M. (2017). Chromosomal localization of *Wolbachia* inserts in the genomes of two subspecies of *Chorthippus parallelus* forming a Pyrenean hybrid zone. *Chrom. Res.* 25, 215-225.**

Zabal-Aguirre, M., Arroyo, F. and Bella, J.L. (2010). Distribution of *Wolbachia* infection in *Chorthippus parallelus* populations within and beyond a Pyrenean hybrid zone. *Heredity* 104, 174-184.
